# Supplementary material for: Machine learning of human plasma lipidomes for obesity estimation in a large population cohort
Source: PLoS Biol. 2019 Oct 18;17(10):e3000443. doi: 10.1371/journal.pbio.3000443 (PMC6799887; doi:10.1371/journal.pbio.3000443)
Supplement: S1 Text — BFP, body fat percentage. (PDF) [file pbio.3000443.s001.pdf]

Supplemental Text: Machine learning of human plasma lipidomes for obesity estimation in a large population cohort

Mathias J. Gerl, Christian Klose, Michal A. Surma, Celine Fernandez, Olle Melander, Satu Männistö, Katja Borodulin, Aki S. Havulinna, Veikko Salomaa, Elina Ikonen, Carlo V. Cannistraci and Kai Simons

S1 Text

Lipid correlations with body fat percentage

A comprehensive Spearman correlation analysis of lipid subspecies and additional features was performed with age as covariable and individually for male and female subjects (S12 Fig). In numerous publications age and sex have been indicated as important covariables in lipidomics studies [1–6]. Male and female show a number of differences in their lipidomes, especially in their TAG and SM classes (S3 Fig, S12 Fig). Overall the correlations of individual lipid subspecies with BFP are in the range of -0.34 to 0.48, with all lipid classes contributing significant correlations (S12A Fig). Male correlations are usually greater than female correlations. Significantly different correlation coefficients between sexes were determined by direct comparison in a statistical test [7] and indicated (S12 Fig). Although we expect females to be largely postmenopausal, with the median age of individuals at 52 years (S2 Table), the lower correlation coefficients in females could be caused by menstrual cycles and the influence of contraceptives [5]. Although we present correlations of the lipidome with BFP, most previous research was done on BMI and obesity [2, 4, 8]. However, BFP and BMI have a monotonic relationship (S2E Fig) and show similar correlation profiles, with lesser correlation coefficients for BMI (S13 Fig). Therefore, BMI related literature will be cited in the following. TAG and DAG correlate especially positively with BFP (S12B Fig). This was universally found for BMI in other studies for all [2, 8] or most [4] significant hits. The TAG and DAG class values as well as many of the male species show significantly greater correlation coefficients than their female counterparts. There is also more DAG and TAG in male plasma [9] (S3 Fig). In general we find highest correlations with TAGs of medium total length (50–52 carbon atoms, S12D Fig) and 1–2 double bonds (S12E Fig). This is consistent with a recent review [10], which described an ensemble of TAGs with total lengths ranging from 48 to 54 and less than two double bonds as *common fatty markers* in multiple diseases involving markers dysregulated lipogenesis. Likely fatty acids for these TAG species are 16:0;0. 16:1;0, 18:0;0 and 18:1;0 [4]. These TAG species have been interpreted as a signature of increased *de novo* synthesis with human fatty acid synthase (FAS), desaturated by stearoyl-CoA-desaturase-1 (SCD-1) and incorporated into TAGs by glycerol-3-phosphate acyltransferase-1 (GPAT-1) [11]. Cholesteryl ester as a class is not significantly correlated with BFP or BMI (S12B Fig) but shows an interesting pattern for lipid species (S12B Fig). The most abundant CE species (CE 18:2;0, CE 18:1;0 and CE 16:0;0) do not or only very weakly correlate with BFP, while there are stronger positive correlations with CE 16:1;0, CE 20:3;0 and CE 20:4;0 and inverse correlations with low abundant CE 20:1;0, CE 20:2;0. A similar pattern for BMI was found before [12]. Weir et al [4] found a strong positive β-coefficients for total CE when regressing BMI and similarly positive coefficients for the major CE species. However, also here CE 20:1;0 is one of the few CE species with an inverse coefficient. PC species are largely positively

correlated, and the fatty acid profile has several similarities to that of CE. Indeed, high correlations of lipid species were found for lipid species of PC and CE if both share the same fatty acid and were attributed to the lecithin-cholesterol acyl transferase (LCAT) enzyme preferably transferring the *sn*-2 position fatty acid from PC to cholesterol [13].

PE is positively correlated with BFP for males, but not for females, in total class and individual species. All ceramides measured are positively correlating with BFP, however a more diverse picture is seen in other publications [4, 8]. A recent publication [14] hypothesized that ceramides serve as gauges of free fatty acid excess when the TAG storage capacity is saturated and the fatty acids spillover into the sphingolipid synthesis pathway. The resulting ceramides initiate cellular responses that prevent membrane dissolution by detergent-like FFAs. PC O- as a whole class and PC O- individual subspecies show some of the greatest inverse correlations. This is most obvious for BMI [2, 4, 8]. Positive slopes have also been found for BMI for similar lipid species, for which we see positive correlations: e.g. PC O-36:4 or PC O-36:5 [4]. PE O- seems to behave very alike PC O- in terms of subspecies abundance, fatty acid abundance and correlations. However, the PE O- class correlation value does not correlate at all with BFP and correlation coefficients are in general not as great as for PC O-. PC O- and PE O- are ether linked to fatty alcohols at the *sn*-1 position. In case of plasmalogen, the vinyl-ether-linked alcohol is particularly susceptible to oxidation [15], which could explain the inverse correlations in situations with high oxidative stress such as obesity [16]. Some LPC correlation for BFP are as low as the PC O- values. LPC has been found numerous times to be reduced with obesity [17, 18] which could be due to LCAT activity as a major source of LPC in circulation. Studies have shown LCAT activity is decreased in obesity [19, 20] potentially leading to a decrease in circulating LPC.

In this dataset glycerophospholipids have been resolved to molecular subspecies level [21], which provides fatty acids information [22]. Although TAG molecular subspecies cannot be resolved, we provide the fatty acid composition behind all overlapping species. This allows to create overall profiles of all for all glycerolipid (GL) and glycerophospholipid (GP) categories measured.

For TAG and sphingolipids (SP) the total saturation and length profiles are examined. In males there is an interesting pattern of SP with total length of 34 are correlated inversely with BFP, while species with total length of 36 are positively correlated (S12F Fig). At species levels there are multiple sex specific correlations, this is in line with the highly significant sex differences in SMs (S3 Fig). e.g. SM 34:1;2 is inversely correlated with BFP in males, while SM 34:2;2 is positively correlated in females with a similar correlation estimate, a pair which has the greatest positive (SM 34:1;2) and negative (SM 34:2;2) lipid β-coefficients in the lasso model. As ceramides, the precursors to SM, are all positively correlated with similar estimates across all species and little sex differences, this is a very interesting effect in sphingomyelin biosynthesis related to obesity.

Measuring enzyme activity is difficult in humans. However, enzyme activity can be estimated by using product-to-precursor ratios under the assumption that omega-6 fatty acids predominate in the contemporary [23]. In all complex lipids measured on the subspecies level, ratios of fatty acids were calculated for the three existing humans desaturases: SCD1/Δ-9-desaturase (SCD-16 (16:1;0/16:0;0), SCD-18 (18:1;0/18:0;0), D9D), Δ-6-desaturase (D6D) and Δ-5-desaturase (D5D) [24–26], two fatty acid elongases (Elongation Of Very Long Chain Fatty Acids Protein 5 (ELOVL5) and 6 (ELOVL6)) [23, 27, 28], as well as an index measuring the de novo lipogenesis (DNL) [29] (S12G Fig, See section "Product-to-precursor ratios" above). The question which lipid classes to include in the indices is not trivial, as fatty acid distribution to lipid classes is very different. Most studies use free fatty acids, while one study also used phospholipids,

resulting in indices that were sometimes quite different from free fatty acid indices [30]. Changes in these indices could reflect dietary intake or altered endogenous PUFAs metabolism. Previous studies found estimated activities of D6D and D9D being positively associated with adiposity [12, 24, 31, 32] while an inverse relation independent of BMI was observed for D5D [12, 25, 31–33]. Also, we see positive correlations for both D9D and the D6D ratio and an inverse correlation for D5D, which is expected as adiposity and triglycerides are correlating with BFP. A study in plasma phospholipids also found positive correlations in SCD-16, D6D and inverse correlations with D5D (while SCD-18 was strongly inversely correlated) [30]. Also, insulin resistance and insulin resistance-related disorders are connected with high activities of the D9D and D6D and a low activity of D5D [25, 27]. It has been suggested that insulin plays a regulatory role in SCD-16 and SCD-18 activity, where insulin increases SCD-16 activity [25]. While we see little correlation of BFP to elongation from 18:3;0 to 20:3;0 (ELOVL5), there is a strong inverse correlation of in the 18:0;0/16;0;0 ratio (ELOVL6). Similarly, a drop in the ELOVL6 ratio have been observed in overweight and obese study participants and was a strong predictor in a model for homeostatic model assessment for insulin resistance (HOMA-IR) [27].

References

1. Trabado S, Al-Salameh A, Croixmarie V, Masson P, Corruble E, Fève B, et al. The human plasma-metabolome: Reference values in 800 French healthy volunteers; impact of cholesterol gender and age. PLOS ONE. 2017;12(3):e0173615. doi:10.1371/journal.pone.0173615.

2. Frahnow T, Osterhoff MA, Hornemann S, Kruse M, Surma MA, Klose C, et al. Heritability and responses to high fat diet of plasma lipidomics in a twin study. Scientific reports. 2017;7(1):3750.

3. Rist MJ, Roth A, Frommherz L, Weinert CH, Krüger R, Merz B, et al. Metabolite patterns predicting sex and age in participants of the Karlsruhe Metabolomics and Nutrition (KarMeN) study. PLOS ONE. 2017;12(8):e0183228. doi:10.1371/journal.pone.0183228.

4. Weir JM, Wong G, Barlow CK, Greeve MA, Kowalczyk A, Almasy L, et al. Plasma lipid profiling in a large population-based cohort. Journal of lipid research. 2013;54(10):2898–2908.

5. Sales S, Graessler J, Ciucci S, Al-Atrib R, Vihervaara T, Schuhmann K, et al. Gender Contraceptives and Individual Metabolic Predisposition Shape a Healthy Plasma Lipidome. Scientific Reports. 2016;6(1). doi:10.1038/srep27710.

6. Huynh K, Barlow CK, Jayawardana KS, Weir JM, Mellett NA, Cinel M, et al. High-Throughput Plasma Lipidomics: Detailed Mapping of the Associations with Cardiometabolic Risk Factors. Cell chemical biology. 2018;.

7. Diedenhofen B, Musch J. cocor: A Comprehensive Solution for the Statistical Comparison of Correlations. PLOS ONE. 2015;10(4):e0121945. doi:10.1371/journal.pone.0121945.

8. Tonks KT, Coster AC, Christopher MJ, Chaudhuri R, Xu A, Gagnon-Bartsch J, et al. Skeletal muscle and plasma lipidomic signatures of insulin resistance and overweight/obesity in humans. Obesity. 2016;24(4):908–916.

9. Seidell JC, Cigolini M, Charzewska J, Ellsinger BM, Björntorp P, Hautvast JG, et al. Fat distribution and gender differences in serum lipids in men and women from four European communities. Atherosclerosis. 1991;87(2-3):203–210.

10. Balgoma D, Pettersson C, Hedeland M. Common Fatty Markers in Diseases with Dysregulated Lipogenesis. Trends in endocrinology and metabolism: TEM. 2019;30(5):283–285.

11. Coleman RA, Mashek DG. Mammalian triacylglycerol metabolism: synthesis, lipolysis, and signaling. Chemical reviews. 2011;111(10):6359–6386.

12. Warensjö E, Öhrvall M, Vessby B. Fatty acid composition and estimated desaturase activities are associated with obesity and lifestyle variables in men and women. Nutrition, metabolism, and cardiovascular diseases : NMCD. 2006;16(2):128–136.

13. Gerl MJ, Vaz WLC, Domingues N, Klose C, Surma MA, Sampaio JL, et al. Cholesterol is Inefficiently Converted to Cholesteryl Esters in the Blood of Cardiovascular Disease Patients. Scientific reports. 2018;8(1):14764.

14. Chaurasia B, Tippetts TS, Mayoral Monibas R, Liu J, Li Y, Wang L, et al. Targeting a ceramide double bond improves insulin resistance and hepatic steatosis. Science (New York, NY). 2019;365(6451):386–392.

15. Lessig J, Fuchs B. Plasmalogens in biological systems: their role in oxidative processes in biological membranes, their contribution to pathological processes and aging and plasmalogen analysis. Current medicinal chemistry. 2009;16(16):2021–2041.

16. Meikle PJ, Summers SA. Sphingolipids and phospholipids in insulin resistance and related metabolic disorders. Nature reviews Endocrinology. 2017;13(2):79–91.

17. Butte NF, Liu Y, Zakeri IF, Mohney RP, Mehta N, Voruganti VS, et al. Global metabolomic profiling targeting childhood obesity in the Hispanic population. The American journal of clinical nutrition. 2015;102(2):256–267.

18. Ho JE, Larson MG, Ghorbani A, Cheng S, Chen MH, Keyes M, et al. Metabolomic Profiles of Body Mass Index in the Framingham Heart Study Reveal Distinct Cardiometabolic Phenotypes. PLoS ONE. 2016;11(2):e0148361.

19. Angell R, Mitsuhashi Y, Bigley K, Bauer JE. Plasma LCAT activity and lipid subfraction composition in obese beagles undergoing weight loss. Lipids. 2009;44(5):415–424.

20. Bajnok L, Seres I, Varga Z, Jeges S, Peti A, Karanyi Z, et al. Relationship of endogenous hyperleptinemia to serum paraoxonase 1, cholesteryl ester transfer protein, and lecithin cholesterol acyltransferase in obese individuals. Metabolism: clinical and experimental. 2007;56(11):1542–1549.

21. Aimo L, Liechti R, Hyka-Nouspikel N, Niknejad A, Gleizes A, Götz L, et al. The SwissLipids knowledgebase for lipid biology. Bioinformatics. 2015;31:2860–6.

22. Surma MA, Herzog R, Vasilj A, Klose C, Christinat N, Morin-Rivron D, et al. An automated shotgun lipidomics platform for high throughput comprehensive, and quantitative analysis of blood plasma intact lipids. European Journal of Lipid Science and Technology. 2015;117(10):1540–1549. doi:10.1002/ejlt.201500145.

23. Kjellqvist S, Klose C, Surma MA, Hindy G, Mollet IG, Johansson A, et al. Identification of Shared and Unique Serum Lipid Profiles in Diabetes Mellitus and Myocardial Infarction. Journal of the American Heart Association. 2016;5(12).

24. Nakamura MT, Nara TY. Structure, function, and dietary regulation of delta6, delta5, and delta9 desaturases. Annual review of nutrition. 2004;24:345–376.

25. Vessby B, Gustafsson IB, Tengblad S, Boberg M, Andersson A. Desaturation and elongation of Fatty acids and insulin action. Ann N Y Acad Sci. 2002;967:183–195.

26. Gieger C, Geistlinger L, Altmaier E, Hrabé de Angelis M, Kronenberg F, Meitinger T, et al. Genetics meets metabolomics: a genome-wide association study of metabolite profiles in human serum. PLoS genetics. 2008;4(11):e1000282.

27. Beccarelli LM, Scherr RE, Newman JW, Borkowska AG, Gray IJ, Linnell JD, et al. Associations Among Fatty Acids, Desaturase and Elongase, and Insulin Resistance in Children. Journal of the American College of Nutrition. 2018;37(1):44–50.

28. Moon YA, Ochoa CR, Mitsche MA, Hammer RE, Horton JD. Deletion of ELOVL6 blocks the synthesis of oleic acid but does not prevent the development of fatty liver or insulin resistance. Journal of lipid research. 2014;55(12):2597–2605.

29. Chong MFF, Hodson L, Bickerton AS, Roberts R, Neville M, Karpe F, et al. Parallel activation of de novo lipogenesis and stearoyl-CoA desaturase activity after 3 d of high-carbohydrate feeding. The American journal of clinical nutrition. 2008;87(4):817–823.

30. Warensjö E, Rosell M, Hellenius ML, Vessby B, de Faire U, Risérus U. Associations between estimated fatty acid desaturase activities in serum lipids and adipose tissue in humans: links to obesity and insulin resistance. Lipids in health and disease. 2009;8(1):37.

31. Pan DA, Hulbert AJ, Storlien LH. Dietary fats, membrane phospholipids and obesity. The Journal of nutrition. 1994;124(9):1555–1565.

32. Warensjö E, Risérus U, Vessby B. Fatty acid composition of serum lipids predicts the development of the metabolic syndrome in men. Diabetologia. 2005;48(10):1999–2005.

33. Steffen LM, Vessby B, Jacobs DR, Steinberger J, Moran A, Hong CP, et al. Serum phospholipid and cholesteryl ester fatty acids and estimated desaturase activities are related to overweight and cardiovascular risk factors in adolescents. International journal of obesity (2005). 2008;32(8):1297–1304.
