# Supplementary figures and images for: Machine learning of human plasma lipidomes for obesity estimation in a large population cohort
Source: PLoS Biol. 2019 Oct 18;17(10):e3000443. doi: 10.1371/journal.pbio.3000443 (PMC6799887; doi:10.1371/journal.pbio.3000443)

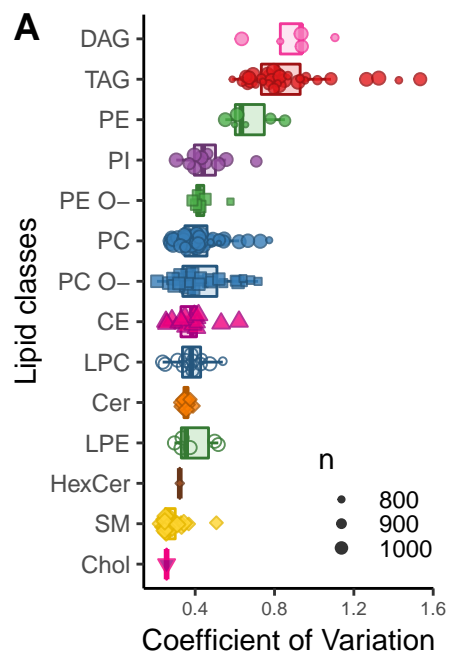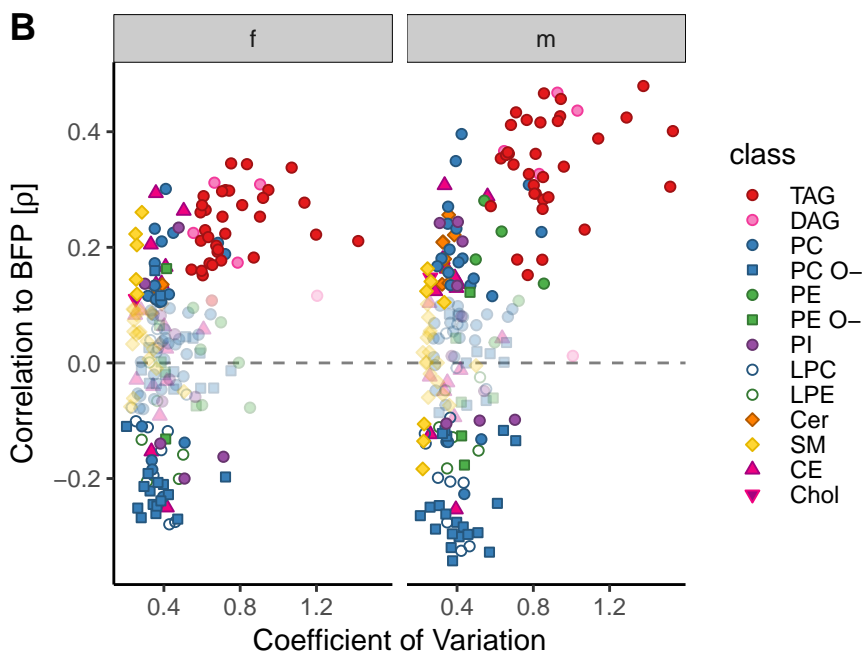

Supplement: S1 Fig — (A) Coefficient of variation (CVi=SDimeani) for all lipid subspecies, clustered and colored by lipid class. The number of measurements per species is indicated as point size (n). (B) Coefficient of variation for all lipid species within each sex individually are shown on the x-axis. On the y-axis Spearman correlations (ρ) to BFP are displayed as in Fig 4 in the main article. Coefficient of variation data is provided in the S3 Table. BFP, body fat percentage. (PDF) [file pbio.3000443.s002.pdf]

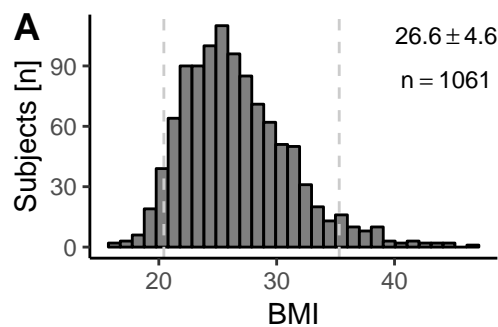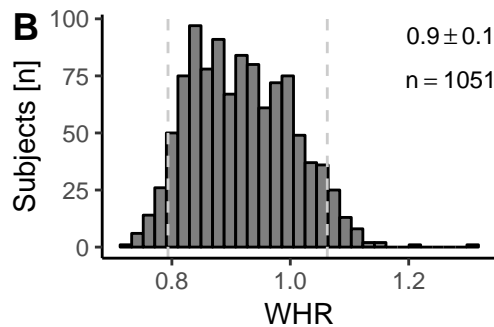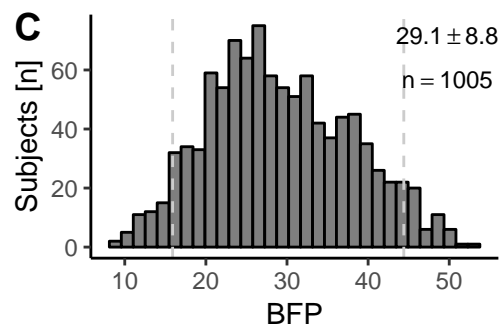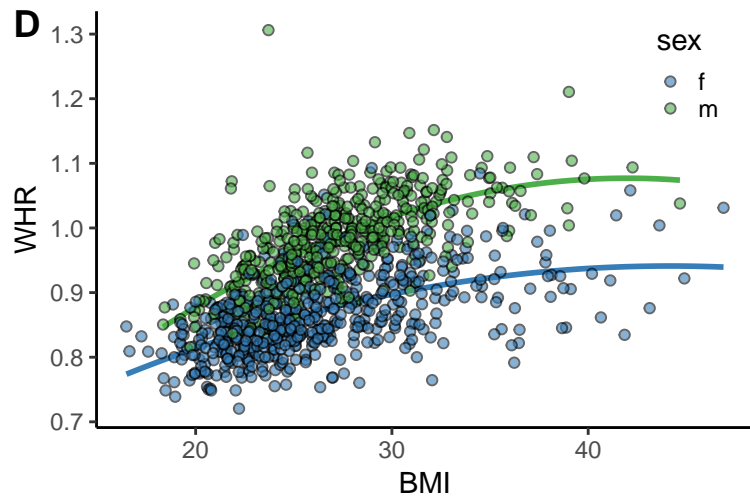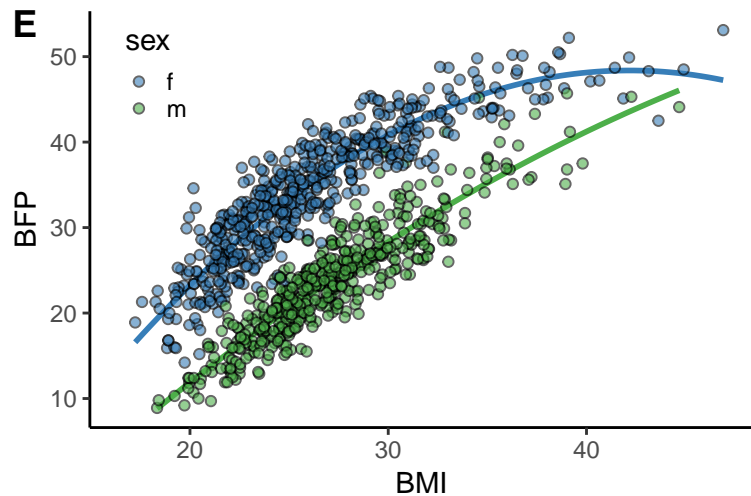

Supplement: S2 Fig — Characteristics of FINRISK variables: Distribution of (A) BMI, (B) WHR, (C) BFPs (Fat mass [%]) in the data set. (A–C) The distribution’s mean, standard deviation, and number of subjects (n), excluding missing values, are shown in the upper left corner. Vertical dashed lines indicate the 5th and 95th percentile. Relationship of BMI to (D) WHR and (E) Fat mass according to subject sex. Lines are based on local polynomial regression fitting (loess). BFP, body fat percentage; BMI, body mass index; WHR, waist-hip ratio. (PDF) [file pbio.3000443.s003.pdf]

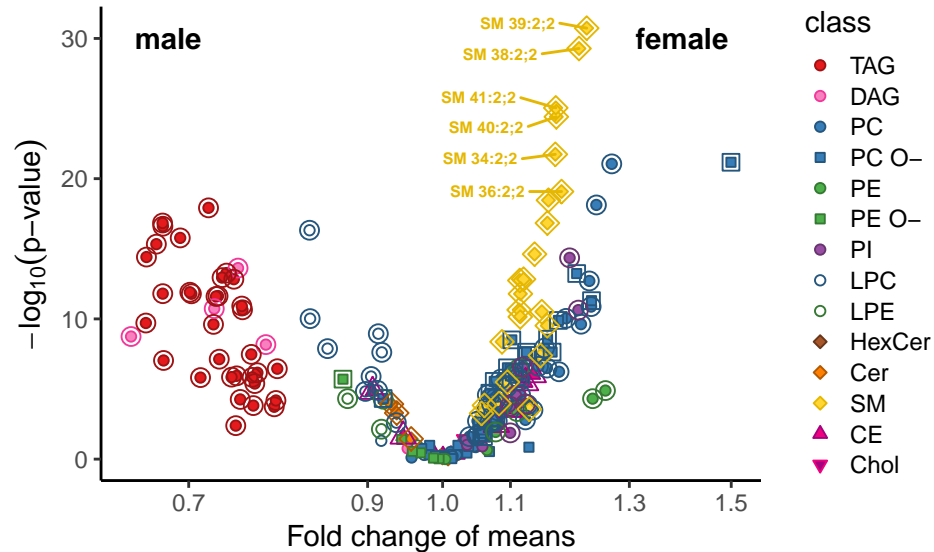

Supplement: S3 Fig — Volcano plot of sex differences between 571 female and 490 male subjects. P-values of the Mann–Whitney U test are displayed on the y-axis, fold changes of means are shown on the x-axis, which is calculated as female (f) divided by male (m) lipid levels (FC=lipid(f)lipid(m)). Labels at the top also indicate the direction of the fold change. Points with outlines show significance <0.05 after Benjamini-Hochberg correction for multiple testing. Some of most significant SM species containing 4E,14Z-sphingadiene are labeled. All data displayed is provided in S4 Table. SM, sphingomyelin. (PDF) [file pbio.3000443.s004.pdf]

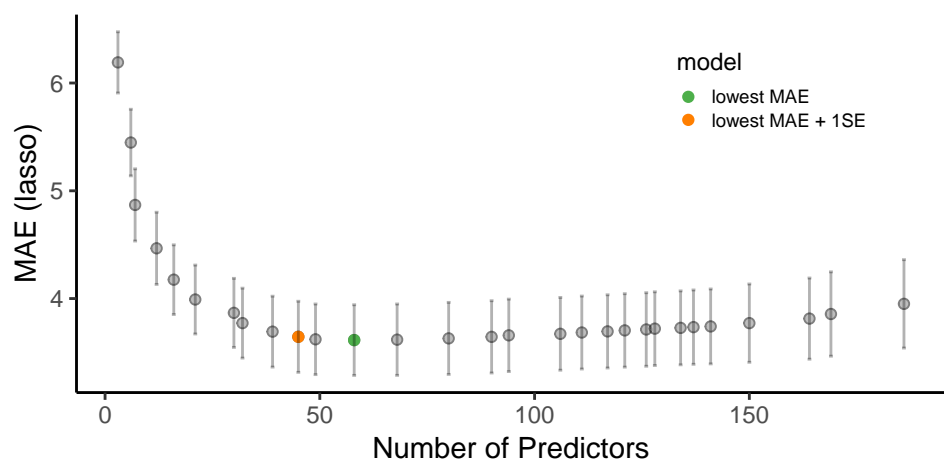

Supplement: S4 Fig — MAE for BFP Lasso models with different number of predictors. The lowest MAE of 3.61 ± 0.33 was achieved by a fraction of 0.24, which used 58 lipid subspecies, whereas a model at 1 standard error distance with a MAE of 3.65 ± 0.33 used a fraction of 0.2 and 45 lipid predictors. The data are shown in S6 Table. BFP, body fat percentage; MAE, mean absolute error. (PDF) [file pbio.3000443.s005.pdf]

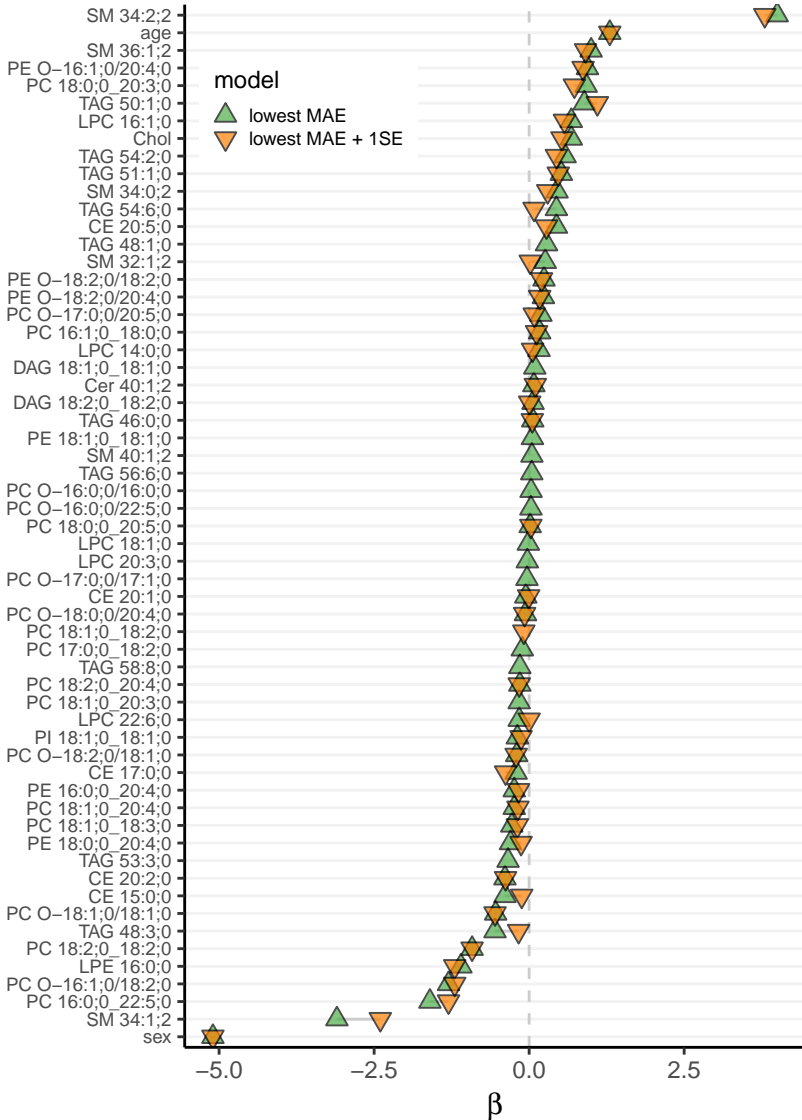

Supplement: S5 Fig — β-coefficients of the predictors of the best Lasso model predicting BFP with the lowest MAE and the model at 1 standard error distance (as in S4 Fig) are displayed. The data are shown in S6 Table. BFP, body fat percentage; MAE, mean absolute error. (PDF) [file pbio.3000443.s006.pdf]

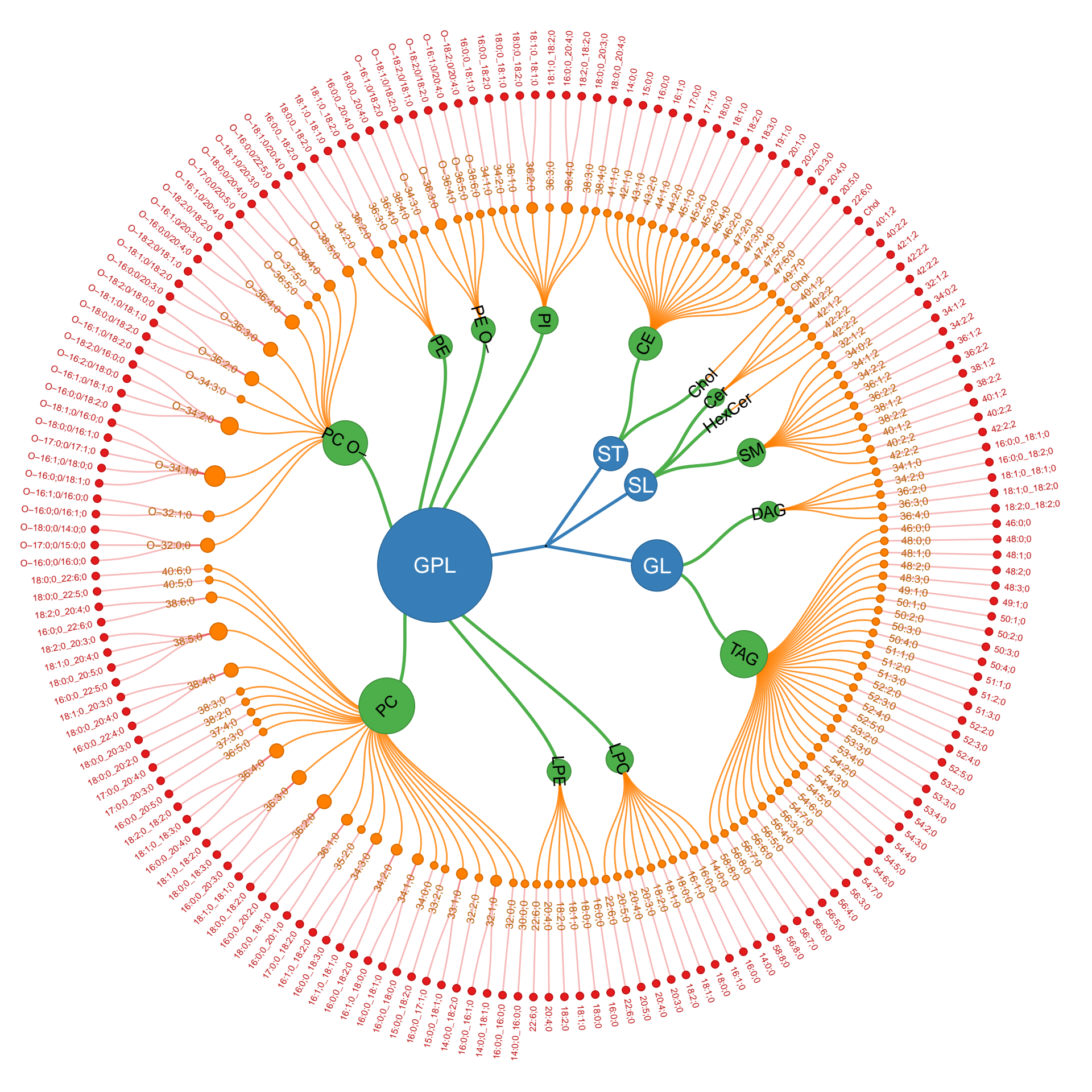

Supplement: S6 Fig — The FINRISK lipidome can be aggregated into 4 categories, 14 lipid classes, 143 species, and 183 lipid species and subspecies. (PDF) [file pbio.3000443.s007.pdf]

**BFP**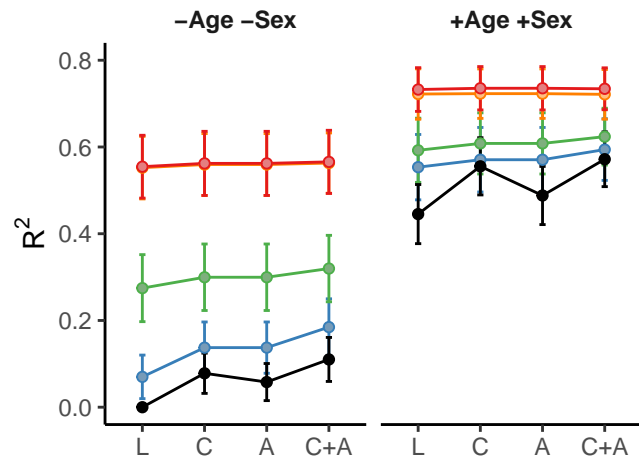**BMI**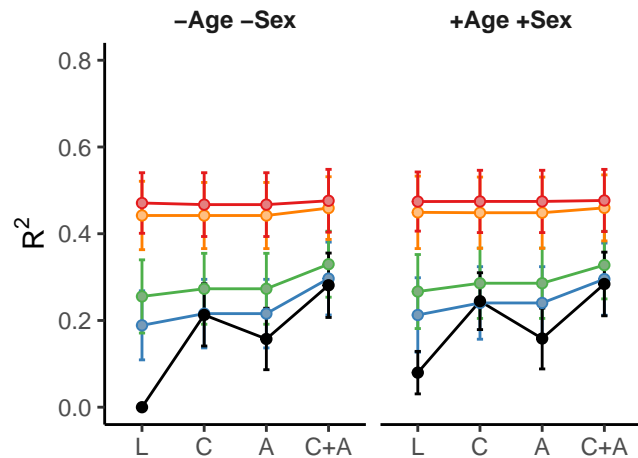**WC**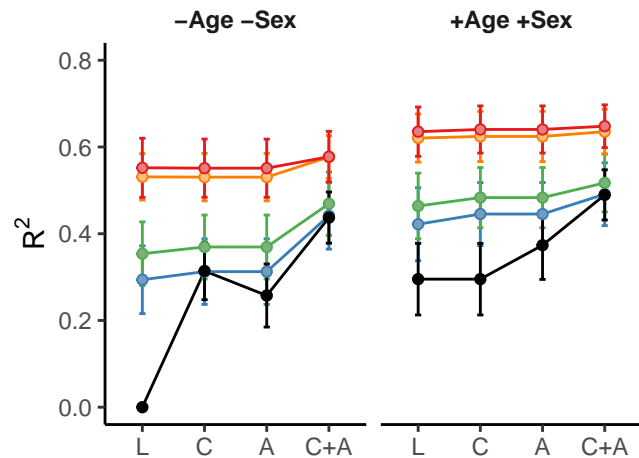**WHR**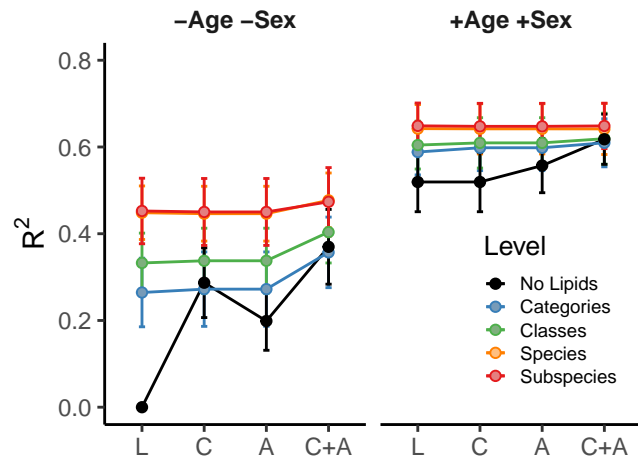

Level

- No Lipids
- Categories
- Classes
- Species
- Subspecies

Supplement: S7 Fig — Effect of different input variables and lipidome detail on the BFP, BMI, WC, and WHR regressions: R2 cross-validation mean and standard deviation (n = 50) based on the FINRISK training data set (n = 805) are shown on the y-axis. Zero models, which always give back the mean of the distribution, were set to 0. Modeling was done either without age and sex as covariables (−Age −Sex) or with (+Age +Sex). Results are colored according to lipidome detail. Either no lipid information was used (No Lipids), or lipidome information was aggregated into lipid categories (Categories), lipid classes (Classes), and lipid species (Species). Subspecies denotes the highest structural resolution possible on the platform with a mix of species and subspecies. Variables in addition to the lipidome input used are shown on the x-axis: No additional input [L]; routine clinical laboratory variables [C]: total cholesterol, HDL cholesterol, LDL cholesterol, triglycerides, HDL to LDL ratio, total cholesterol to HDL ratio, triglycerides to HDL ratio; additional variables [A]: Blood pressure treatment, lipid treatment, smoker, pregnant, fasting, prevalent diabetes, prevalent CVD, prevalent liver disease, prevalent coronary heart disease, prevalent stroke, systolic blood pressure, diastolic blood pressure; or the combination of clinical and additional variables [C + A]. Special points are the zero model (L, No Lipids, −Age, −Sex), which does not use any predictors but returns the mean of the respective obesity measure variable, or the regression only based on age and sex, (L, No Lipids, +Age, +Sex), both of which are used as references predictability without regression. Values are listed in S9 Table. BFP, body fat percentage; BMI, body mass index; HDL, high-density lipoprotein; LDL, low-density lipoprotein; WC, waist circumference; WHR, waist-hip ratio. (PDF) [file pbio.3000443.s008.pdf]

**A** **Classes removed**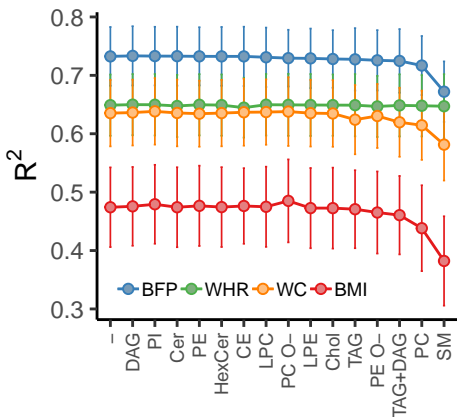**B** **BFP**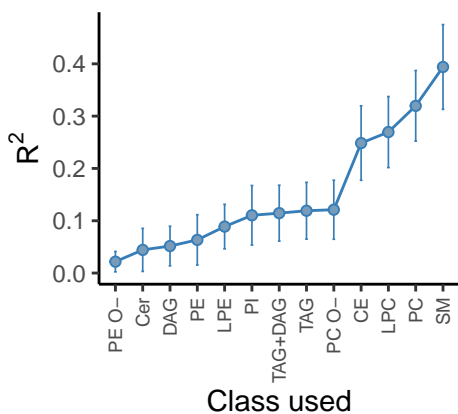**C** **WHR, WC**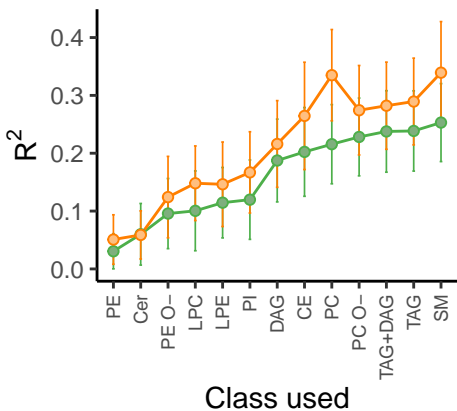**D** **BMI**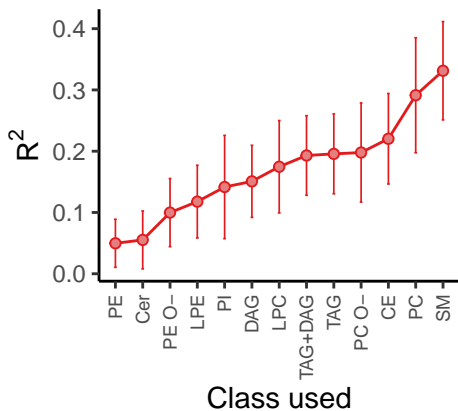

Supplement: S8 Fig — (A) Lasso models for BFP, WHR, WC, and BMI were built on the full data set plus age and sex (indicated by −), or the lipids of the indicated class were removed (indicated by the class). R2 values are shown and sorted by decreasing BFP results. (B–D) Lasso models for BFP, WHR, and BMI were built on the lipids of one lipid class only plus age and sex. R2 values are shown and sorted by increasing results of the respective variable. As TAG and DAG are very correlated, both were also removed together. BFP, body fat percentage; BMI, body mass index; DAG, diacylglyceride; TAG, triacylglyceride; WC, waist circumference; WHR, waist-hip ratio. (PDF) [file pbio.3000443.s009.pdf]

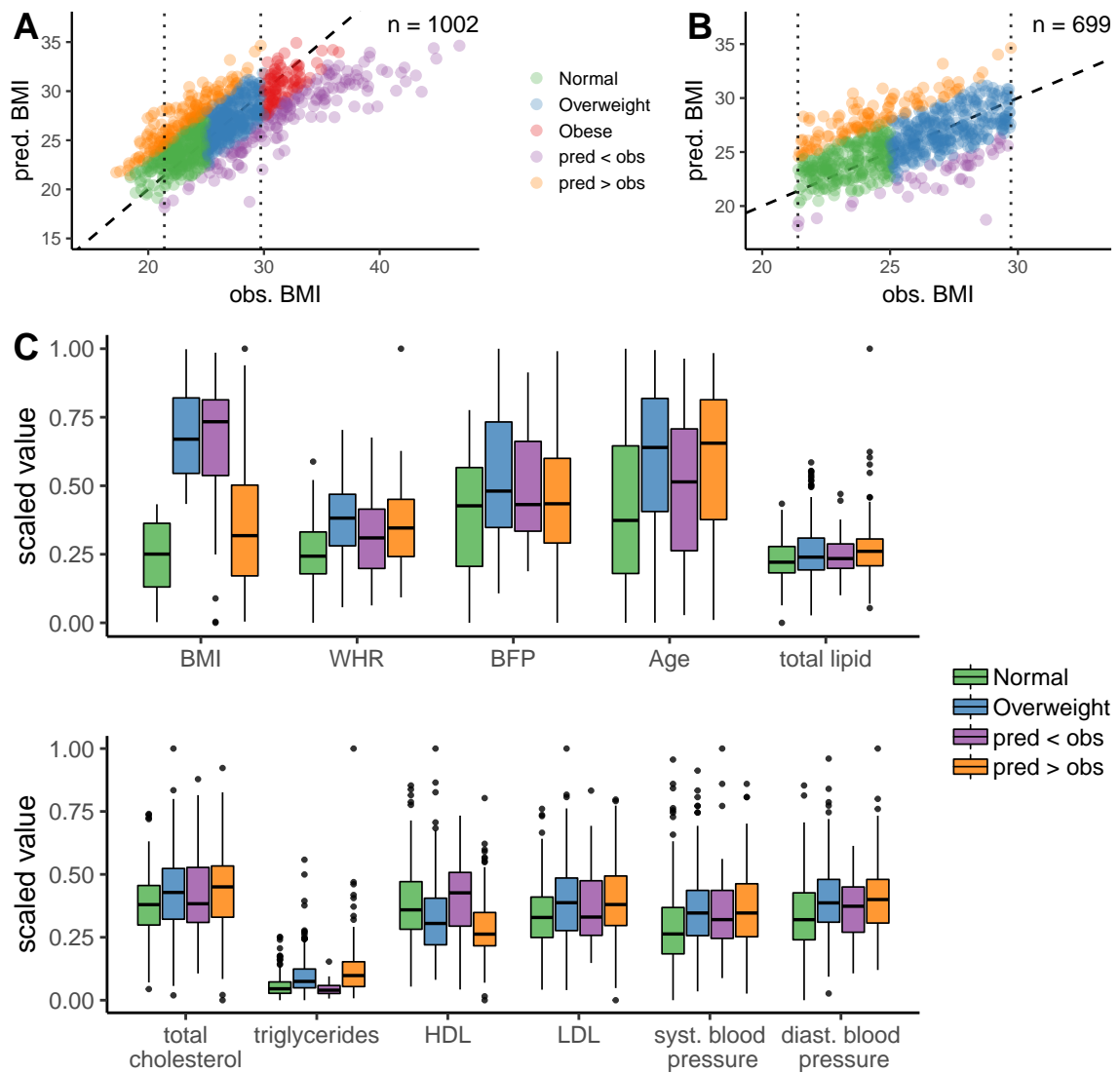

Supplement: S9 Fig — (A) Scatter plot of predicted BMI versus observed BMI. The dashed diagonal line represents a predicted BMI perfectly matching the observed BMI. Individual samples are shown as a scatter plot. Outliers are defined as the upper or lower 15% of the residual distribution. n shows the number of FINRISK samples used for this analysis. Samples, which are not classified as outliers, are colored as Normal (BMI < 25), Overweight (25 ≥ BMI < 30), or Obese (BMI ≥ 25). Horizontal dotted lines indicate samples used for analysis. (B) Samples used for analysis: Because samples classified as "pred < obs" and "pred > obs" are not evenly distributed along the observed BMI axis; the analysis is restricted to observed BMI 21.4 to 29.73, using the lower value of the obs. BMI of the "pred < obs" and the upper value of the "pred > obs" samples as borders for the restricted area. This selects a total 699 of 1,002 (69.8%) samples, with 242 Normal, 311 Overweight, 104 "pred > obs," and 42 "pred < obs" samples. (C) Plotted variables are scaled to a common scale ranging from 0 to 1 and shown as a box plot. Outliers are plotted as black points. BMI, body mass index; obs, observed; pred, predicted. (PDF) [file pbio.3000443.s010.pdf]

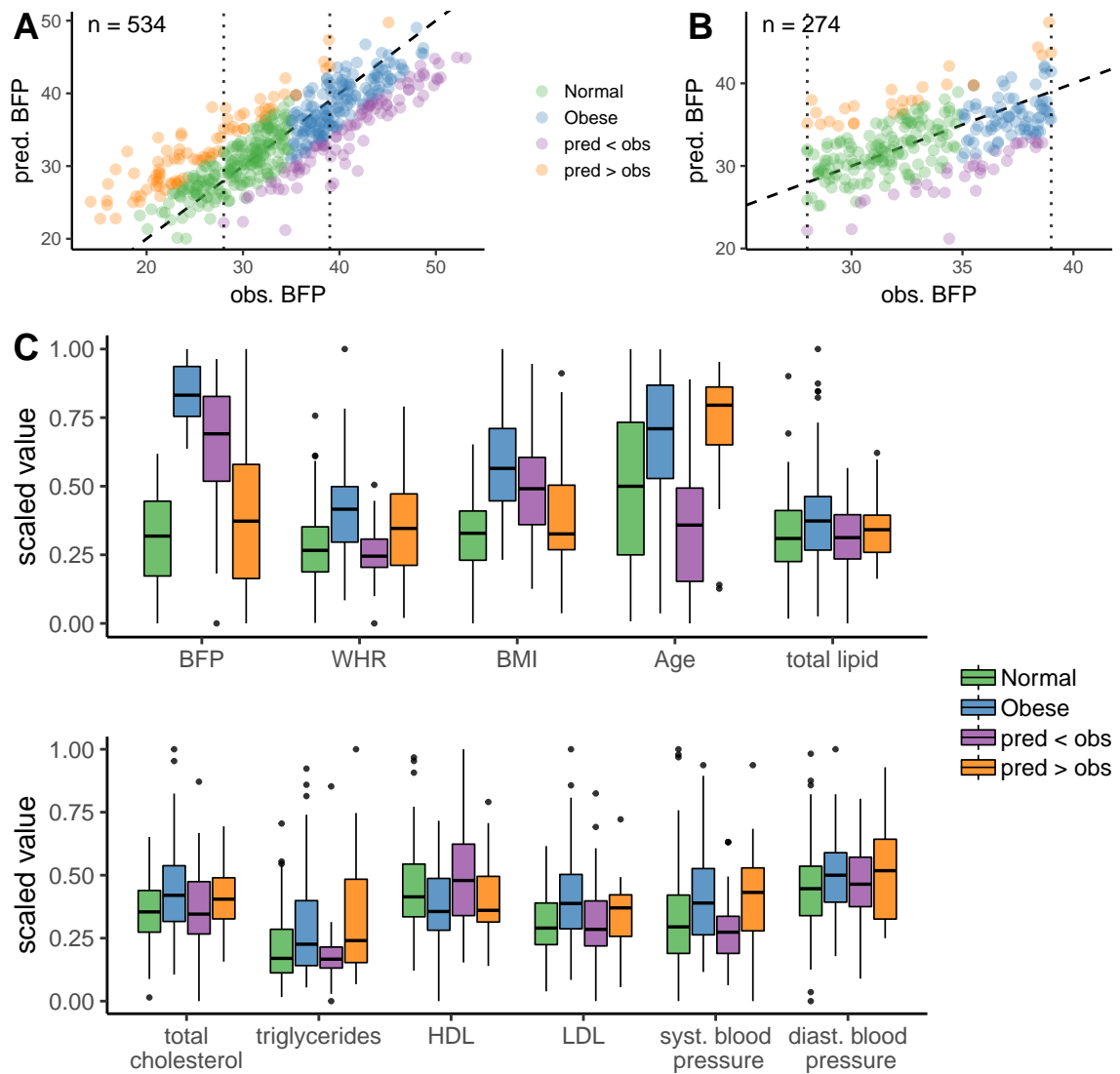

Supplement: S10 Fig — (A) Scatter plot of predicted BFP versus observed BFP. The dashed diagonal line represents a predicted BFP perfectly matching the observed BFP. Individual samples are shown as scatter plot. Outliers are defined as the upper or lower 15% of the residual distribution. n shows the number of FINRISK samples used for this analysis. Samples, which are not classified as outliers, are colored as Normal (BFP < 35) or Obese (BFP ≥ 35) [71, 72]. Horizontal dotted lines indicate samples used for analysis. (B) Samples used for analysis: Because samples classified as "pred < obs" and "pred > obs" are not evenly distributed along the observed BFP axis, the analysis is restricted to observed BFP 28 to 39, using the lower value of the obs. BFP of the "pred < obs" and the upper value of the "pred > obs" samples as approximate guides for the restricted area. The "pred > obs" value beyond obs. BFP of 40 was classified as too extreme and ignored. This selects a total 274 of 534 (51.3%) samples, with 141 Normal, 74 Overweight, 26 "pred > obs," and 33 "pred < obs" samples. (C) Plotted variables are scaled to a common scale ranging from 0 to 1 and shown as a box plot. Outliers are plotted as black points. BFP, body fat percentage; obs, observed; pred, predicted. (PDF) [file pbio.3000443.s011.pdf]

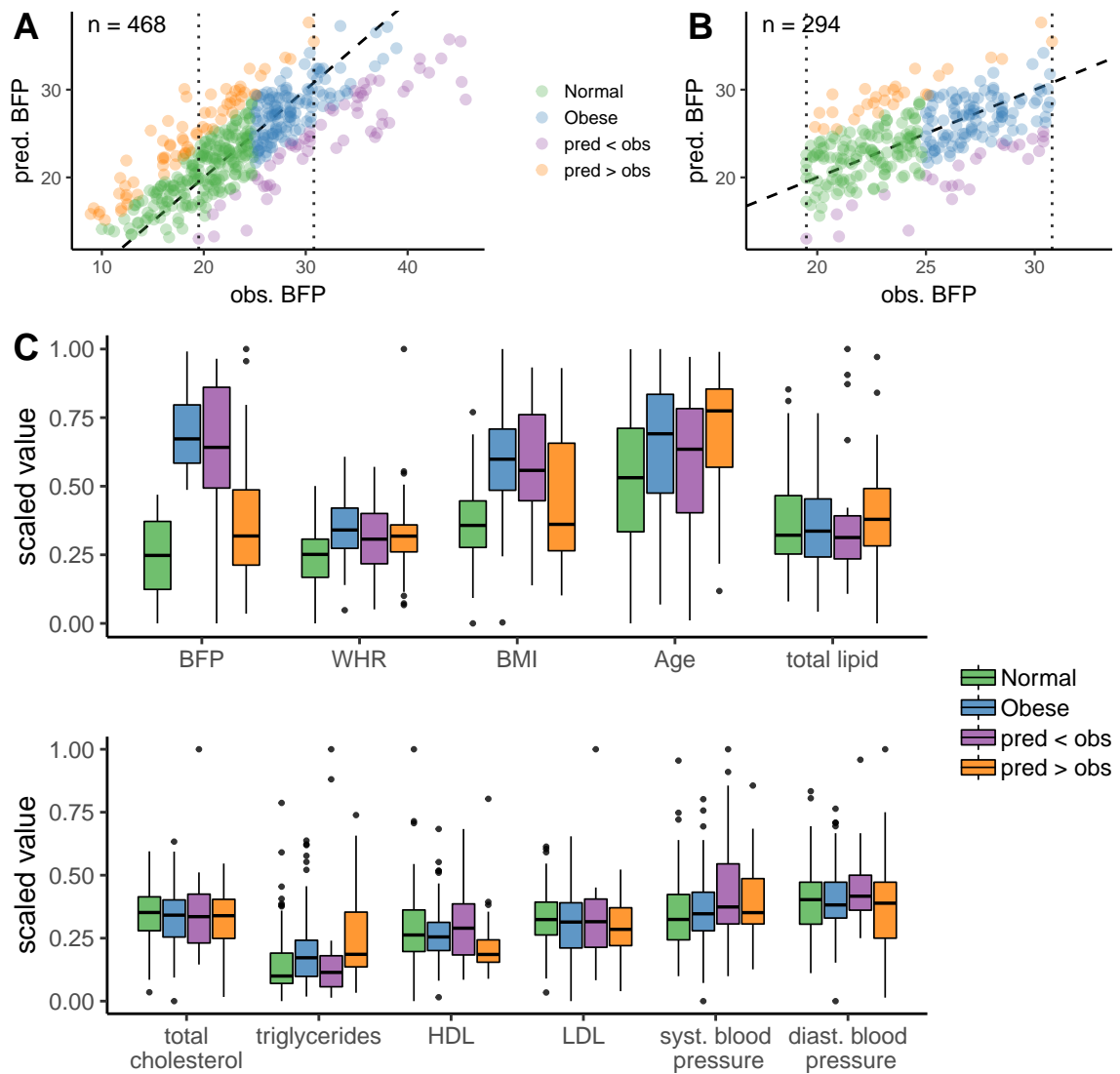

Supplement: S11 Fig — (A) Scatter plot of predicted BFP versus observed BFP. The dashed diagonal line represents a predicted BFP perfectly matching the observed BFP. Individual samples are shown as scatter plot. Outliers are defined as the upper or lower 15% of the residual distribution. n shows the number of FINRISK samples used for this analysis. Samples, which are not classified as outliers, are colored as Normal (BFP < 25) or Obese (BFP ≥ 25) [71, 72]. Horizontal dotted lines indicate samples used for analysis. (B) Samples used for analysis: Because samples classified as "pred < obs" and "pred > obs" are not evenly distributed along the observed BFP axis, the analysis is restricted to observed BFP 19.5 to 30.8, using the lower value of the obs. BFP of the "pred < obs" and the upper value of the "pred > obs" samples as approximate guides for the restricted area. This selects a total 294 of 468 (62.8%) samples, with 134 Normal, 105 Overweight, 29 "pred > obs," and 26 "pred < obs" samples. (C) Plotted variables are scaled to a common scale ranging from 0 to 1 and shown as a box plot. Outliers are plotted as black points. BFP, body fat percentage; obs, observed; pred, predicted. (PDF) [file pbio.3000443.s012.pdf]

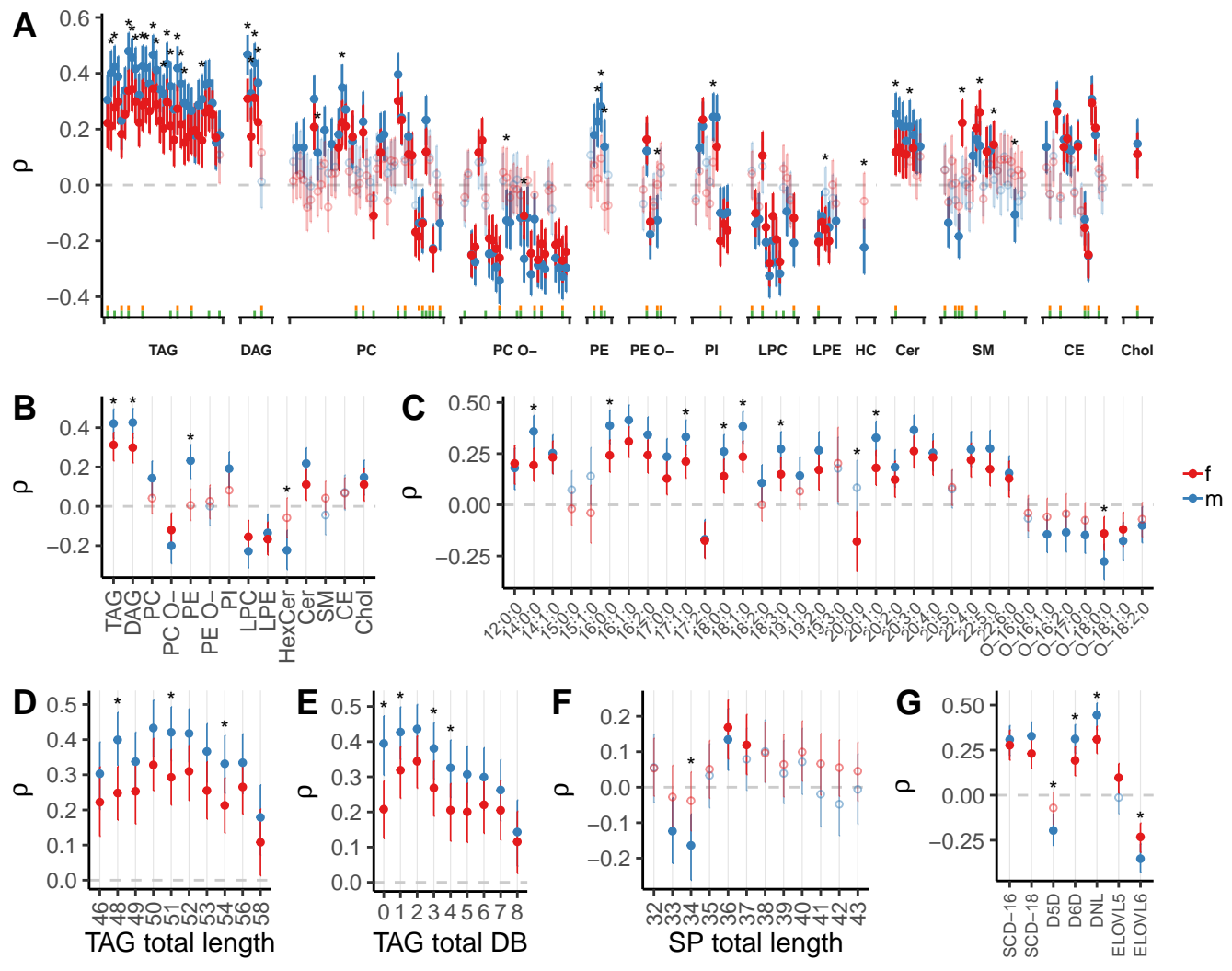

Supplement: S12 Fig — Spearman correlation coefficients (ρ) and their 95% CI for each sex (male and female) and adjusted for subject age are shown for (A) lipid subspecies; HC signifies the HexCer lipid class. Green ticks at the bottom indicate that this subspecies was used in the model with the lowest MAE, whereas yellow ticks indicate the model with 1 standard error from the lowest MAE (S4 Fig). A total number of 202 lipid subspecies were tested in 1,005 subjects, of those 53.5% (108) were significantly correlated with BFP in females and 65.3% (132) in males. (B) class sums; (C) fatty acid sums within the lipid classes CE, DAG, TAG, LPC, LPE, PC, PC O-, PE, PE O-, PI; (D) sums of TAG total lengths; (E) sums of TAG total double bonds; (F) sums of sphingolipid total lengths; (G) Product-to-precursor fatty acid ratios for SCD1/ Δ-9-desaturase (SCD-16, SCD-18), Δ-6-desaturase (D6D), Δ-5-desaturase (D5D), fatty acid elongases ELOVL5 and ELOVL6, and DNL index. Correlations with Benjamini-Hochberg corrected p < 0.05 are shown with filled points, whereas correlations with p > 0.05 are shown with open circles and transparently. Differences between male and female correlations were compared, and significant differences are indicated by an asterisk (*). Results are provided in S11 Table. BFP, body fat percentage; CE, cholesteryl ester; DAG, diacylglyceride; LPC, lysophosphatidylcholine; LPE, lysophosphatidylethanolamine; MAE, mean absolute error; PC, phosphatidylcholine; PC O-, 1-O-alkyl- or 1-O-alkenyl-phosphatidylcholine; PE, phosphatidylethanolamine; PE O-, 1-O-alkyl- or 1-O-alkenyl-phosphatidylethanolamine; PI, phosphatidylinositol; TAG, triacylglyceride. (PDF) [file pbio.3000443.s013.pdf]

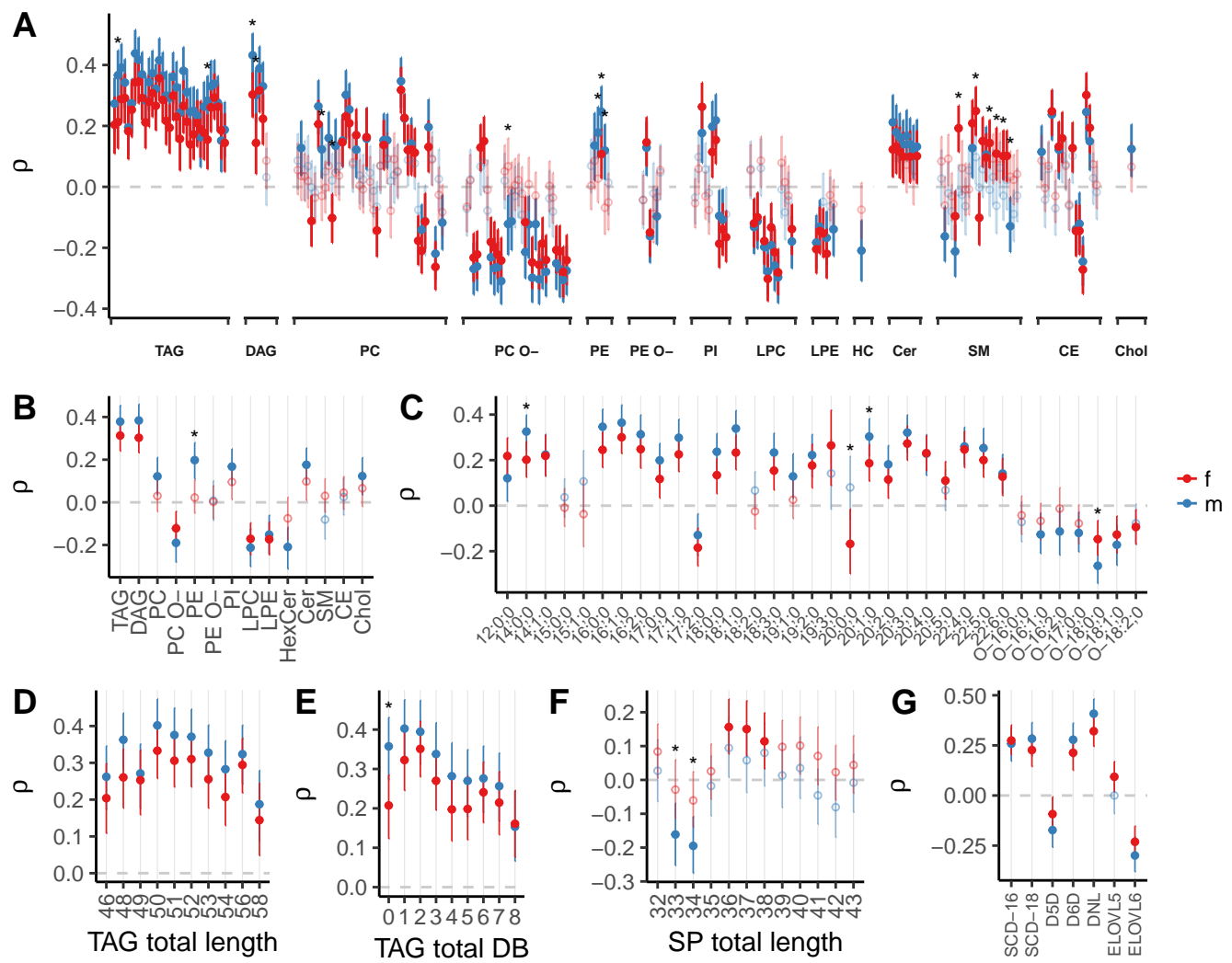

Supplement: S13 Fig — Spearman correlation coefficients (ρ) and their 95% CI for each sex (male and female) and adjusted for subject age are shown for (A) lipid subspecies; HC signifies the HexCer lipid class. A total number of 202 lipid subspecies were tested in 1,061 subjects, of those 59.9% (121) were significantly correlated with BMI in females and 60.4% (122) in males. (B) Class sums; (C) fatty acid sums within the lipid classes CE, DAG, TAG, LPC, LPE, PC, PC O-, PE, PE O-, PI; (D) sums of TAG total lengths; (E) sums of TAG total double bonds; (F) sums of sphingolipid total lengths; (G) Product-to-precursor fatty acid ratios for SCD1/Δ-9-desaturase (SCD-16, SCD-18), Δ-6-desaturase (D6D), Δ-5-desaturase (D5D), fatty acid elongases ELOVL5 and ELOVL6, and DNL index. Correlations with Benjamini-Hochberg corrected p < 0.05 are shown with filled points, whereas correlations with p > 0.05 are shown with open circles and transparently. Differences between male and female correlations were compared and significant differences are indicated by an asterisk (*). Results are provided in S11 Table. BMI, body mass index; CE, cholesteryl ester; DAG, diacylglyceride; LPC, lysophosphatidylcholine; LPE, lysophosphatidylethanolamine; MAE, mean absolute error; PC, phosphatidylcholine; PC O-, 1-O-alkyl- or 1-O-alkenyl-phosphatidylcholine; PE, phosphatidylethanolamine; PE O-, 1-O-alkyl- or 1-O-alkenyl-phosphatidylethanolamine; PI, phosphatidylinositol; TAG, triacylglyceride. (PDF) [file pbio.3000443.s014.pdf]

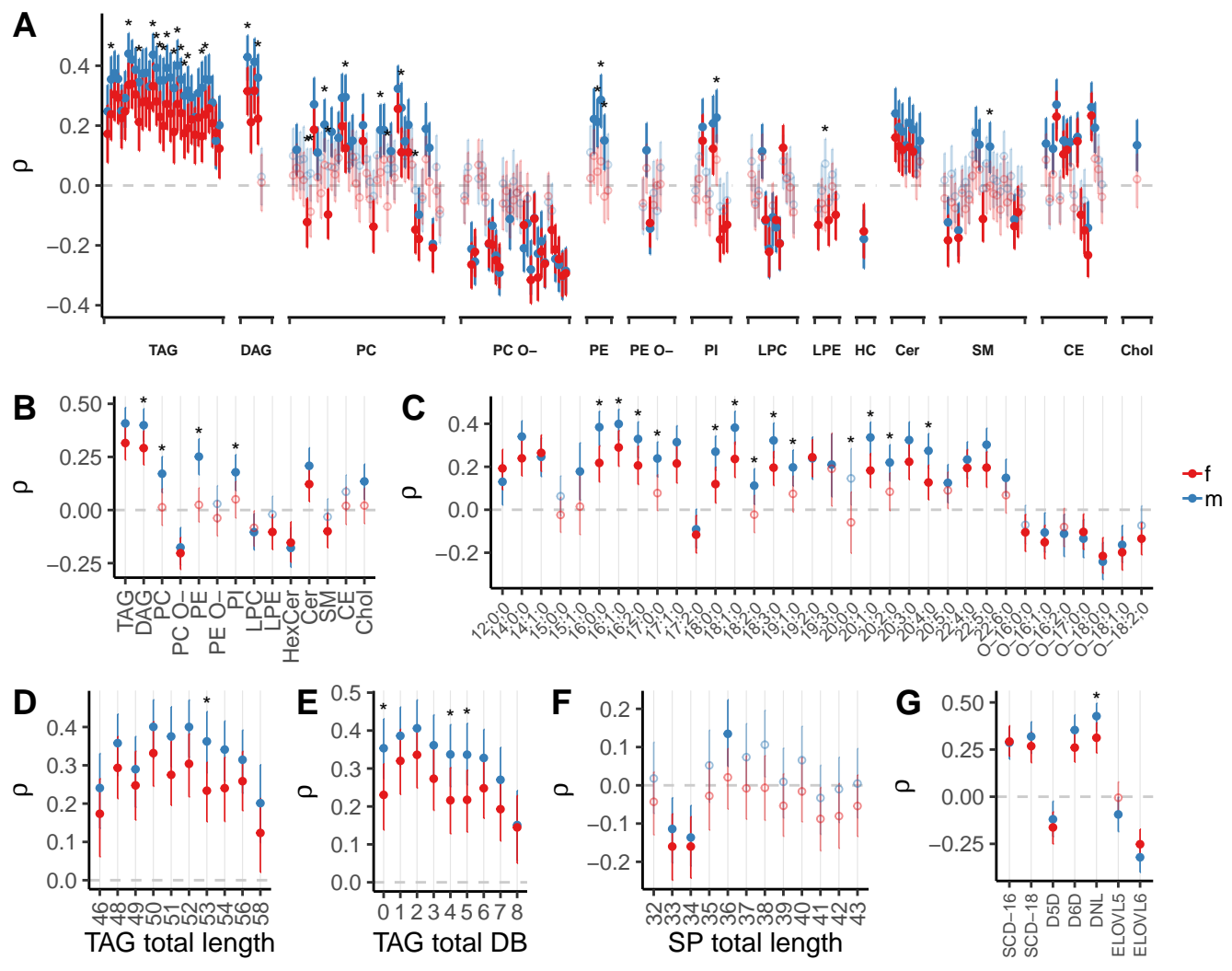

Supplement: S14 Fig — Spearman correlation coefficients (ρ) and their 95% CI for each sex (male and female) and adjusted for subject age are shown for (A) lipid subspecies; HC signifies the HexCer lipid class. A total number of 202 lipid subspecies were tested in 1,051 subjects, of those 49.5% (100) were significantly correlated with WHR in females and 58.4% (118) in males. (B) Class sums; (C) fatty acid sums within the lipid classes CE, DAG, TAG, LPC, LPE, PC, PC O-, PE, PE O-, PI; (D) sums of TAG total lengths; (E) sums of TAG total double bonds; (F) sums of sphingolipid total lengths; (G) Product-to-precursor fatty acid ratios for SCD1/Δ-9-desaturase (SCD-16, SCD-18), Δ-6-desaturase (D6D), Δ-5-desaturase (D5D), fatty acid elongases ELOVL5 and ELOVL6, and DNL index. Correlations with Benjamini-Hochberg corrected p < 0.05 are shown with filled points, whereas correlations with p > 0.05 are shown with open circles and transparently. Differences between male and female correlations were compared and significant differences are indicated by an asterisk (*). Results are provided in S11 Table. CE, cholesteryl ester; DAG, diacylglyceride; LPC, lysophosphatidylcholine; LPE, lysophosphatidylethanolamine; MAE, mean absolute error; PC, phosphatidylcholine; PC O-, 1-O-alkyl- or 1-O-alkenyl-phosphatidylcholine; PE, phosphatidylethanolamine; PE O-, 1-O-alkyl- or 1-O-alkenyl-phosphatidylethanolamine; PI, phosphatidylinositol; TAG, triacylglyceride; WHR, waist-hip ratio. (PDF) [file pbio.3000443.s015.pdf]

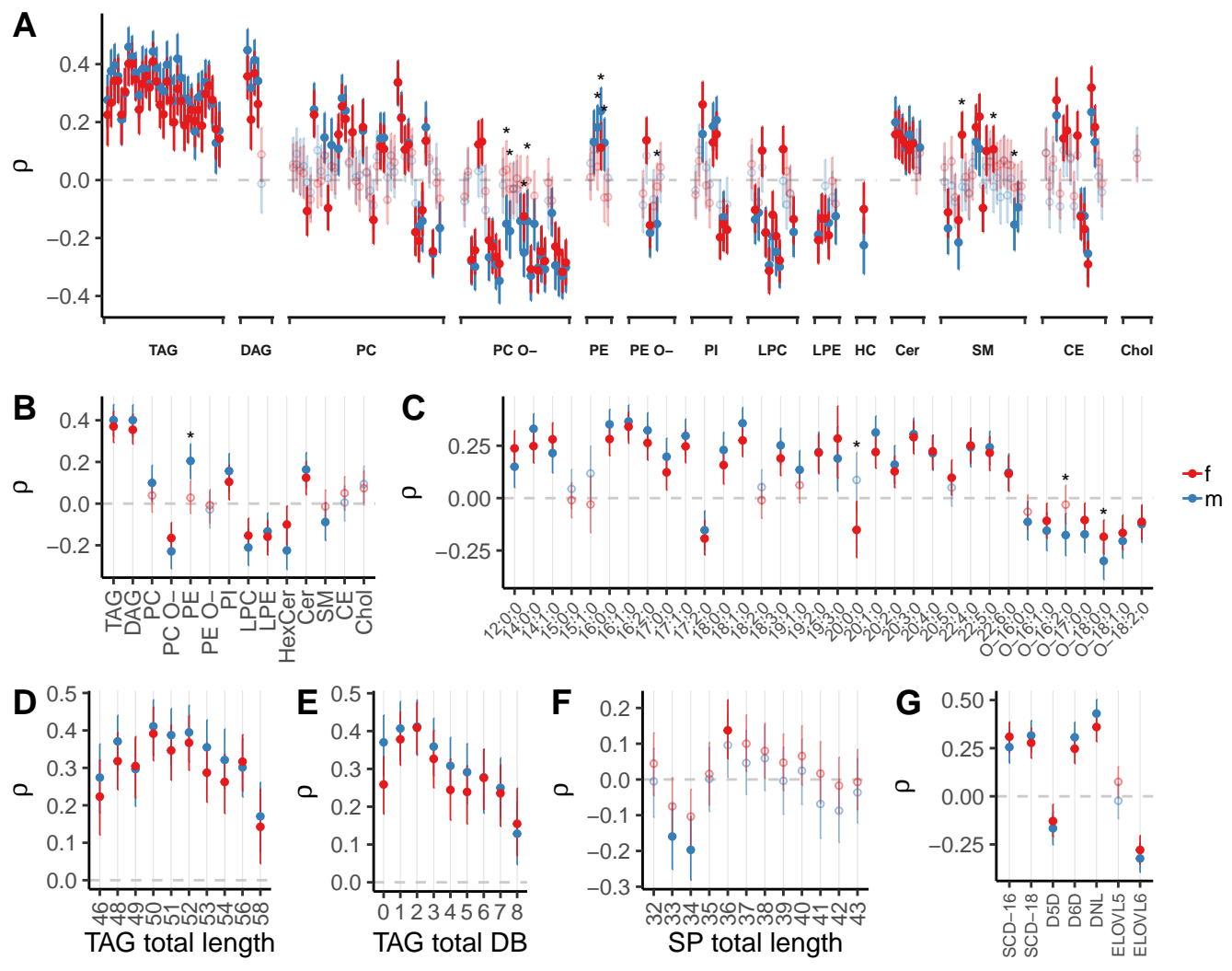

Supplement: S15 Fig — Spearman correlation coefficients (ρ) and their 95% CI for each sex (male and female) and adjusted for subject age are shown for (A) lipid subspecies; HC signifies the HexCer lipid class. A total number of 202 lipid subspecies were tested in 1,052 subjects, of those 59.4% (120) were significantly correlated with WC in females and 57.9% (117) in males. (B) class sums; (C) fatty acid sums within the lipid classes CE, DAG, TAG, LPC, LPE, PC, PC O-, PE, PE O-, PI; (D) sums of TAG total lengths; (E) sums of TAG total double bonds; (F) sums of sphingolipid total lengths; (G) Product-to-precursor fatty acid ratios for SCD1/Δ-9-desaturase (SCD-16, SCD-18), Δ-6-desaturase (D6D), Δ-5-desaturase (D5D), fatty acid elongases ELOVL5 and ELOVL6, and DNL index. Correlations with Benjamini-Hochberg corrected p < 0.05 are shown with filled points, whereas correlations with p > 0.05 are shown with open circles and transparently. Differences between male and female correlations were compared and significant differences are indicated by an asterisk (*). Results are provided in S11 Table. CE, cholesteryl ester; DAG, diacylglyceride; LPC, lysophosphatidylcholine; LPE, lysophosphatidylethanolamine; MAE, mean absolute error; PC, phosphatidylcholine; PC O-, 1-O-alkyl- or 1-O-alkenyl-phosphatidylcholine; PE, phosphatidylethanolamine; PE O-, 1-O-alkyl- or 1-O-alkenyl- phosphatidylethanolamine; PI, phosphatidylinositol; TAG, triacylglyceride; WC, waist circumference. (PDF) [file pbio.3000443.s016.pdf]

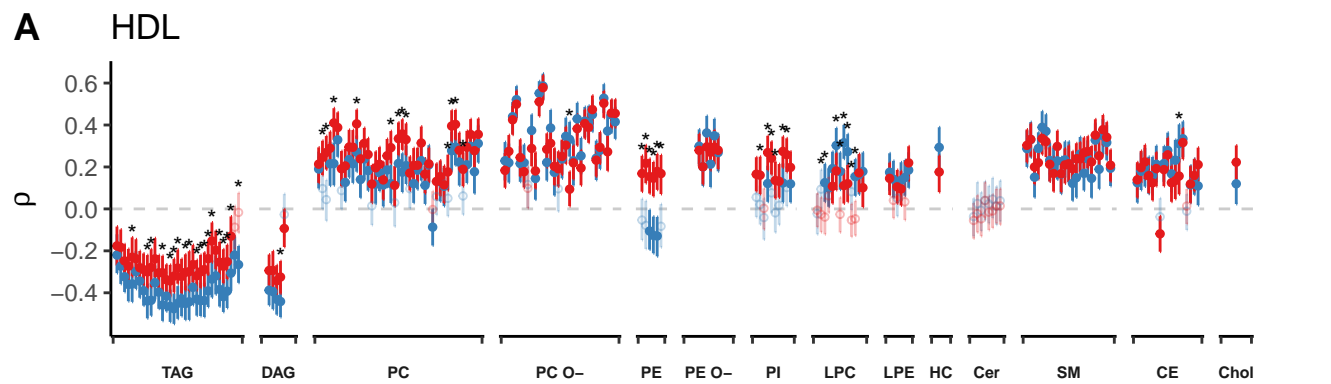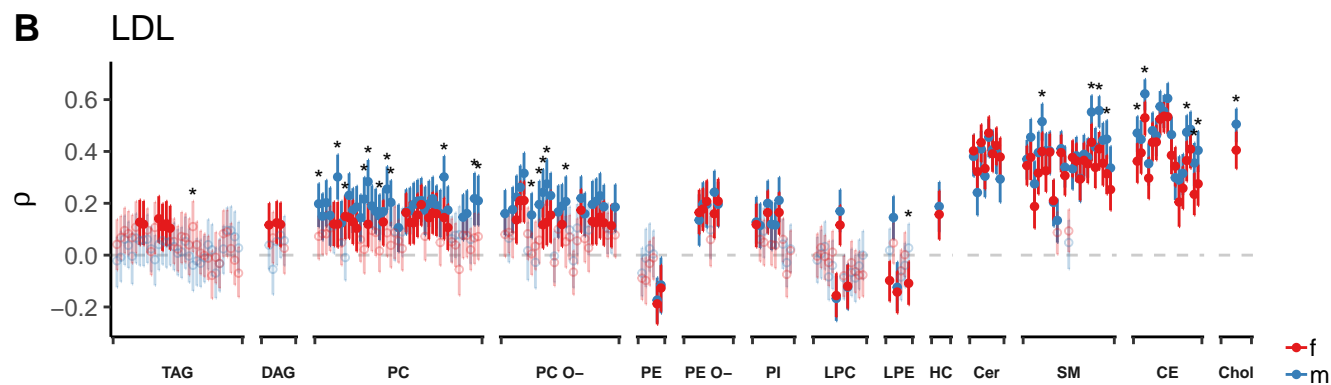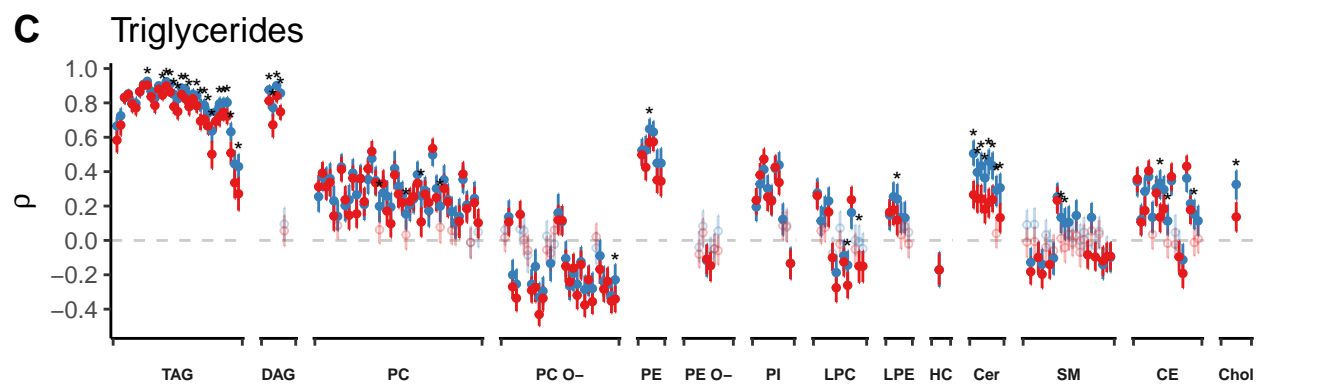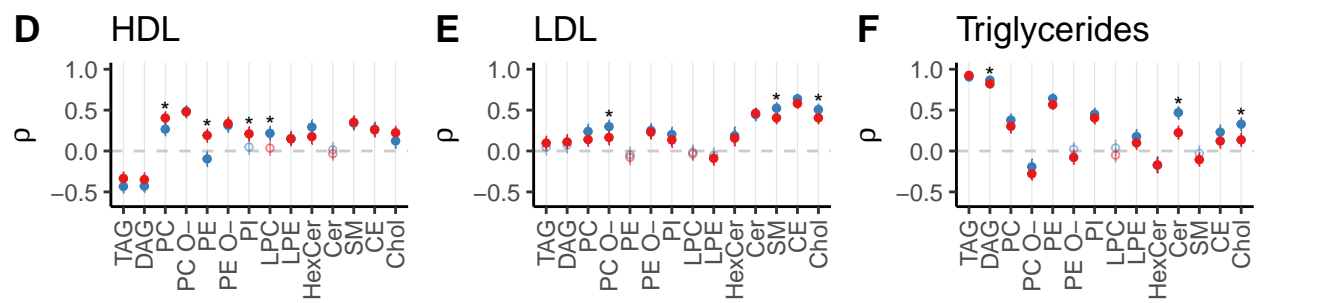

Supplement: S16 Fig — Spearman correlation coefficients (ρ) and their 95% CI for each sex (male and female) and adjusted for subject age are shown for lipid species and (A) HDL, (B) LDL, and (C) triglycerides, as well as lipid class sums, and (D) HDL, (E) LDL, and (F) triglycerides; correlations with Benjamini-Hochberg corrected p < 0.05 are shown with filled points, whereas correlations with p > 0.05 are shown with open circles and transparently. Differences between male and female correlations were compared, and significant differences are indicated by an asterisk (*). HDL, high-density lipoprotein; LDL, low-density lipoprotein. (PDF) [file pbio.3000443.s017.pdf]
