# Supplementary material for: Machine learning of human plasma lipidomes for obesity estimation in a large population cohort
Source: PLoS Biol. 2019 Oct 18;17(10):e3000443. doi: 10.1371/journal.pbio.3000443 (PMC6799887; doi:10.1371/journal.pbio.3000443)
Supplement: Attachment — Submitted filename: Response_to_Reviewers.pdf [file pbio.3000443.s029.pdf]

Point-by-point response to all of the reviewers' comments

We thank the editor and reviewers for their helpful comments. The point-by-point response to each comment are provided below, along with the text modifications and line numbers made in the revised manuscript based on the suggestions.

****
Reviews

Reviewer #1: Jens Nielsen, signed review

This is a very interesting paper describing the use of shut-gun lipidomics for profiling of obese subjects. Lipidomics data are used together with traditional measurements such as BMI, WC and WHR to identify a subset of lipid measurements that can be used to predict phenotypes. The authors use non-linear regression analysis (machine learning) for this analysis, and the derived model is shown to have good predictive strength. I think there are particular two interesting findings from the study: 1) that a small set of biomarkers are not sufficient to predict and capture the complex phenotypes in obese subjects, and 2) through a validation cohort the it is found that the exact timing of fasting is not influencing the set of biomarkers.

I only have one major comment and one minor comment.

Major comment:
There is no discussion of the possible molecular mechanisms associated with the biomarkers. The authors could look into whether these are mainly diet associated or are they associated with certain features, e.g. inflammation. I know it will be hard to have a detailed mechanistic discussion/explanation, but some insight along this line would significantly enrich the paper.

Thank you for your comment. Please see the answer to your minor comment for details on this question

Minor comment:
It is stated that the 45 lipid species in the reduced model are essentially the same as the 58 lipid species in the better model. I suggest the authors quantify this statement instead of just saying essentially. Why not give the exact overlap in the lipid species in the two models. This could also be used in the discussion I am requesting on mechanisms, as overlapping lipid species are likely associated with some sort of mechanisms.

Thank you for your valuable suggestion. We took the opportunity to look the different predictors in more detail. We now list the predictors and beta-coefficients of both Lasso models in Table S6 and visualize them is a separate supplemental Figure S5A. This way it is clearly shown, which predictor is used by which model. In addition, we built a Pearson

correlation network of the predictors (Figure S5B) and used the structure of the network to discuss molecular details of the predictors in the manuscript as follows:

=========================== line 299
The Pearson correlation network of the predictors of both Lasso models (S5B Fig) shows several interesting features. Within the common lipid predictors of both BFP Lasso models, SM 34:1;2 has the greatest negative and SM 34:2;2 the greatest positive lipid β-coefficients by far (S5A Fig, S6 Table), although both are highly correlated in the correlation network within a cluster of other SM species. The additional double bond in SM 34:2;2 is very likely due to an 18;2;2 long-chain base [57,58], which is abundant in human plasma [58] and has been shown to be a 4E,14Z-sphingadiene [59]. SM 34:2;2 and further doubly unsaturated SMs correlate positively with BFP, i.e. SM 36:2;2 and SM 38:2;2 especially in males (S11 Table). In contrast to their correlations these lipids show higher levels in females (S3 Fig, S4 Table, [27]). The 4E,14Z-sphingadiene is suggested to be produced by an unknown desaturase, which also creates the single 14Z double bond in 1-deoxysphingolipids [60]. Its supposed higher activity in females results in higher levels of the respective Cers and SMs [27]. Here the occurence of 4E,14Z-sphingadiene in specifically SMs is the major correlation with BFP picked up by the Lasso models. Their importance is supported by the importance of the SM class for predictions (S8 Fig) and the fact the SMs are a particularly stable lipid class in plasma (S1 Fig). How the balance between sphingosine and 4E,14Z-sphingadiene is mechanistically related to the overall metabolic status and its usefulness as a general BFP biomarker needs to be further investigated. However, if 4E,14Z-sphingadiene is correlated with BFP independent of sex, the differences in its levels between the sexes might be simply explained by higher BFP in females in general (S2E Fig).
Associated with the SM cluster are multiple lipid predictors (CE 15:0;0, CE 17:0;0 and PC O-17:0;0/17:1;0) with odd chain fatty acids, which could be due to dairy consumption [61] or dietary fiber intake [62]. However, their association with SM and Cer species (S5B Fig) might also indicate that these fatty acids are derived from hydroxylated fatty acids in glycosphingolipids or phytosphingosine [63] and therefore link the model to sphingolipids not measured in this study. Furthermore, we find a cluster of correlated lyso-lipids and of TAG species (S5B Fig). TAGs with positive β-coefficients are largely consistent with common lipid biomarkers [64]. A more detailed discussion of this observation and the association of product-to-precursor ratios of lipid metabolism enzymes to obesity measures is provided in the S1 Text. ==========================

In addition we referenced and updated supplementary text S1 named “Lipid correlations with body fat percentage”, in which we discuss additional molecular details. We already had moved this part from the actual manuscript to the supplemental text to make the paper more focussed.

Reviewer #2:

The authors used two sets of lipidomics data ("1061 participants of the FINRISK 2012 population cohort" and "250 randomly chosen participants of the Malmö Diet and Cancer Cardiovascular Cohort") to build and test the regression model for predicting body mass index (BMI), waist circumference (WC), waist-hip ratio (WHR) and body fat percentage (BFP) with consideration of gender and age. In the conclusion, the authors conclude that "we can use machine learning to model and validate obesity estimates better than by using classical clinical parameters and find lipid specific differences between the individual estimates." My major concern is that as the conventional standard of obesity such as the "body mass index (BMI), waist circumference (WC), waist-hip ratio (WHR) and body fat percentage (BFP)" are easier to obtain and measure, whether the authors can demonstrate additional benefits of this model besides finding small lipid molecules associated with the obesity indicators.

Thank you for this critical comment.
We updated the following sections as indicated to make our intentions more clear:

Abstract:
============================
Moreover we used this regression to probe the clinically relevant information contained in the plasma lipidome and found that the plasma lipidome also contains information about body fat distribution, since WHR ($R2 = 0.65$) was predicted more accurately than BMI ($R2 = 0.47$).
============================

Author summary:
============================
Here we show that in combination with machine intelligence modeling, lipidomic measurements contain rich information about body fat amount and distribution beyond traditional clinical assays in two human cohorts with a total of 1311 individuals. This result suggests different aspects of lipid metabolism involved in both body fat content and distribution. Investigation of outliers of the regression model reveal important insights into molecular lipid metabolism beyond classical obesity measurements. These findings have implications in the frame of the current debate on metabolically healthy obese individuals. Although the plasma lipidome is influenced by diet, we find the obesity predictions to be independent of fasting duration. To achieve these results, a multi-parametric analysis is required at lipid species resolution and the predicting set of biomarkers has to be sufficiently large.
============================

Introduction:
============================ line 72
Analysing the plasma lipidome or the metabolome [26] to estimate obesity is of course much more complicated than by direct measurement and not what we aimed for. Instead, we are investigating how the plasma lipids reflect metabolic status and whether the plasma lipidome can be used to predict health and disease. There is already ample evidence that the plasma lipidome is changing in different disease states [16,27] and here we show that

the plasma lipidome indeed gives information beyond obesity measures and classical clinical lipid parameters such as triglycerides and cholesterol. We find that the lipidome gives information about the body fat distribution as measured by the WHR because a number of lipid species correlate with the WHR, even when controlled for BMI. Lipidomes show differences between the sexes, concerning lipid levels, lipid coefficients of variation and correlations of lipid species with obesity measures. These correlation profiles were similar between the three obesity estimates, but very different from those lipids correlating with HDL-, LDL-cholesterol, and triglyceride levels indicating that these commonly used lipid markers only insufficiently capture molecular lipid metabolism. We discuss correlations with obesity measures and find that highest lipid impact on our modeling algorithm features 4E,14Z-sphingadiene containing sphingomyelins. Finally, we look the variation not explained by the BMI and BFP regression and find those related to other clinical parameters, such as HDL- and LDL-cholesterol.
============================

Other comments:
1: Page 7 “Five different models predicting BFP were trained and their parameters learned on 796 random training samples in a cross-validation loop (Fig. 1B, Results for WHR and BMI in Table S2).” There are 6 methods in Figure 1B, not 5.

Thank you. The manuscript has been corrected accordingly (line 280).

2: Figure 1C. When comparing the two groups, please perform statistical test(s).

Thank you. We performed a statistical test confirming the two standardizations are not significantly different and updated the figure caption with the result.

============================ Fig 1
“Lasso based NMAE of BFP comparing direct molar amounts (pmol) to molar amounts standardized to the total lipid amount within a sample (mol%). A two-sided, unpaired Mann-Whitney U test resulted in a p-value of 0.99.”
============================

We also tested the significance of the slope in Fig. 1F:

============================ Fig 1
“Histogram of fasting times of subjects in the FINRISK dataset and scatter plot of the Lasso residuals against fasting time, including a linear model. The slope of the linear model had a p-value of 0.33.”
============================

3: Figure 2B. As age and gender have strong weight in the model is very obvious, you can build a model of age and gender separately to see how important it is.

Thank you for your comment. We had already built this model is part of Fig 2. as the "regression only based on age and sex, (L, No Lipids, +Age +Sex)". We slightly change the manuscript text and figure legend to make this more obvious.

=========================== Fig 2
"Special points are the zero model (L, No Lipids, $-$Age $-$Sex), which does not use any predictors but returns the mean of the BFP variable, and the regression only based on age and sex, (L, No Lipids, $+$Age $+$Sex), both of which are used as references for BFP predictability without regression based on L, C or A input (Table S3).
===========================

=========================== line 458
BFP predictions based on age and sex variables alone, already have a MAE of 4.5--5.2 or 45--57% variation explained (Fig. 2, +Age +Sex).
===========================

4: Figure S3. The meaning of the X axis is not clear, which side represents Male/Female?

We updated the text within the figure to read "male" or female and updated the figure caption to make the associations clear.

=========================== Fig S3
"Volcano plot of sex differences between 571 female and 490 male subjects.
P-values of the Mann-Whitney U test are displayed on the y-axis, fold changes of means are shown on the x-axis, which is calculated as female (f) divided by male (m) lipid levels (FC = lipid(f) / lipid(m)). Labels at the top also indicate the direction of the fold change."
===========================

5: Figure S8B, S9B, S10B. How to choose the restricted area? What is the percentage of samples selected as a whole sample? And the restricted area is close to the middle, but a large number of outliers are not in this range.

Thank you for the critical comment. We used the opportunity to refine the selection of the restricted area. This also changes the numbers slightly, but the outcome is unchanged.
As we try to create a situation, which improves the outlier asymmetry, by restricting the area such that both upper and lower outlier are both present at the same time. This inevitable results in a situation, where we only look at parts of the outliers. Please note the figure numbers have shifted

============================ Figure S9

(B) Samples used for analysis: As samples classified as “pred < obs” and “pred > obs” are not evenly distributed along the observed BMI axis, the analysis is restricted to observed BMI 21.4–29.73, using the lower value of the obs. BMI of the “pred < obs” and the upper value of the “pred > obs” samples as borders for the restricted area. This selects a total 699 of 1002 (69.8%) samples, with 242 Normal, 311 Overweight, 104 “pred > obs” and 42 “pred < obs” samples.

============================

============================Figure S10

(B) Samples used for analysis: As samples classified as “pred < obs” and “pred > obs” are not evenly distributed along the observed BFP axis, the analysis is restricted to observed BFP 28–39, using the lower value of the obs. BFP of the “pred < obs” and the upper value of the “pred > obs” samples as approximate guides for the restricted area. The “pred > obs” value beyond obs. BFP of 40 was classified as too extreme and ignored. This selects a total 274 of 534 (51.3%) samples, with 141 Normal, 74 Overweight, 26 “pred > obs” and 33 “pred < obs” samples.

============================

============================Figure S11

(B) Samples used for analysis: As samples classified as “pred < obs” and “pred > obs” are not evenly distributed along the observed BFP axis, the analysis is restricted to observed BFP 19.5–30.8, using the lower value of the obs. BFP of the “pred < obs” and the upper value of the “pred > obs” samples as approximate guides for the restricted area. This selects a total 294 of 468 (62.8%) samples, with 134 Normal, 105 Overweight, 29 “pred > obs” and 26 “pred < obs” samples.

============================

6: Table S2. Please add the test set and validation set results.

We added the additional results to the table, which is now S7 Table.

============================ Table S7

Reproducibility of all models on all obesity estimates using MAE, NMAE and R^2 as metrics: Models were trained on the FINRISK training dataset in a 5x repeated 10-fold cross-validation loop, which results in a cross-validation error. Fitting the models on all the training data, using the best performing parameter set, results in a training error, while testing the models on the hold out test data gives the testing error, and applying the model to the independent Malmö dataset results in the validation error. Only subjects have been used, for which all obesity measures were available (n = 796).

============================

Reviewer #3:

Gerl et al employed a novel mass spectrometric shotgun approach and measured the levels of 183 plasma lipid species in participants of FINRISK 2012 population cohort comprising 1061 plasma samples. Based on this data, authors performed advanced machine learning using different predictive models and identified the association of lipid profile and information about body fat amount and distribution. The conclusion was further validated using an independent dataset of the Malmo Diet and Cancer Cardiovascular Cohort comprising randomly selected 250 plasma lipidomes. It is an interesting study but there are some concerns.

1. Authors need to better explain the merit of this study. Are lipid predictors predict development of obesity at the moment of in future? The study would be of great value if lipid predictors were measured before individuals developed obesity yet, but it would be of little value if lipid predictors simply separate obese and non-obese individuals at the moment, which wouldn't need complex lipid measurement and modelling.

We changed the manuscript in multiple places to highlight the benefits of the study. Please see our response to Reviewer #2.

2. Line 109, "Each batch was accompanied by a set of 4 blank samples (150 mM ammonium bicarbonate (in water)". It is not clear why 150 mM ammonium bicarbonate is chosen as blank samples instead of reconstitution solvent "7.5 mM ammonium acetate in chloroform/methanol/propanol".

Before extraction, plasma samples are diluted 1:50 in 150 mM ammonium bicarbonate (ABC). 180 µl of this dilution are used for extraction with 810 µl of MTBE/MeOH 7:2. In case of blank samples, 180 µl of pure ABC (w/o plasma added) are used for extraction. That is, the blank samples underwent the same extraction, reconstitution and analysis procedure as the actual study samples and thus cover the entire process. Using only the reconstitution solvent as a blank sample would control only the reconstitution step with respect to possible cross-contaminations etc.

3. In method section, line 118 the authors described "Internal standards were pre-mixed with the organic solvent mixture. " More details are needed to assess the robustness of this method. For instance, are internal standards pre-mixed with the organic solvent mixture right before each batch? Organic solvent used for lipid extraction is very volatile so it is challenging to have internal standards in such solvent for long term with consistent concentrations. In addition, what is the volume of organic solvent mixture used in this study? It is generally challenging to consistently transfer small amount of organic solvent due to the less retention on pipette tips.

In order to prevent changes in internal standard concentrations due to unwanted evaporation of solvent, internal standard mix is mixed with the organic extraction mixture (MTBE/MeOH 7:2) only shortly (ca 30 min) before the extraction is started. Additionally, cold solvents (4˚C) are used and flasks are kept tightly closed at all times. In order to ensure accurate pipetting, positive displacement pipettes are used to transfer the internal standard mixture. Pipettes are calibrated regularly by an external and accredited institution. For

accurately pipetting extraction solvent (mixed with internal standards) on the Hamilton starlet pipetting robot, anti-droplet control is enabled, which reliable prevents dripping of organic solvents from the pipette tip. Furthermore, mixing internal standard with the extraction solvent in a master mix for ca 100-200 samples renders pipetting small volumes (<100 µl) unnecessary.

4. In MS data acquisition section, “both MS and MSMS data were combined to 136 monitor CE, DAG and TAG ions as ammonium adducts”. The authors bypassed LC chromatography. It is unclear how authors dealt with in-source fragmentation issues. For instance, TAG would contribute to DAG signals through in-source fragmentation. PC would contribute to lyso PC signals due to in-source fragmentation. Without LC separation, it is hard to tell how authors distinguish these species.

In-source fragmentation is indeed an important aspect in direct infusion mass spectrometry. In positive ion mode, TAG molecules are detected as NH4+ adducts. Similarly, DAG molecules are detected as NH4+ adducts. However, TAG would fragment per neutral loss of a fatty acid plus NH4+ (see figure Figure 1). Therefore, fragments formed upon in-source fragmentation of TAG, would not overlap with NH4+ adducts of DAG and hence not affect quantification of DAG. In order to test whether there is in-source fragmentation of TAG per neutral loss of fatty acid plus NH4+, a TAG standard was infused under the MS settings used in our plasma method. Neutral losses could be detected only at very low amounts, if at all, with intensities amounting to <<0.01% of the precursor intensities (Table 1).

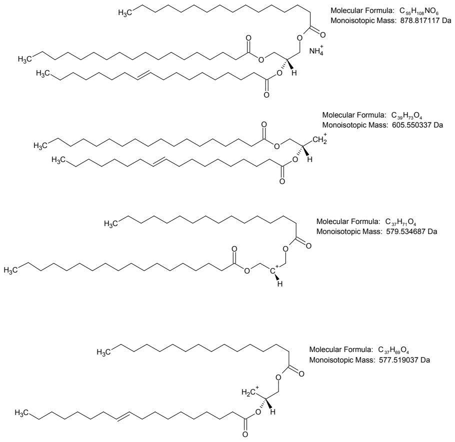

Figure 1: Fragmentation of TAG in positive ion mode.

Table 1: In source fragmentation of a TAG standard.

	Kind	mz	intensity	% of precursor
**TAG 16:0/18:0/18:1**	precursor	878.817	330000000	100
**DAG 16:0/18:1 NL**	fragment	577.519		0
**DAG 16:0/18:0 NL**	fragment	579.535	4200	0.001273
**DAG 18:0/18:1 NL**	fragment	605.55	5400	0.001636

PC molecules are detected in negative ion mode as acetate adducts. Similarly, LPC molecules are detected in negative ion mode as acetate adducts. Upon fragmentation, fatty acid fragments are formed (Figure 2). LPC fragments are typically not formed. Therefore, possible in-source fragmentation of PC would not contribute to LPC signals.

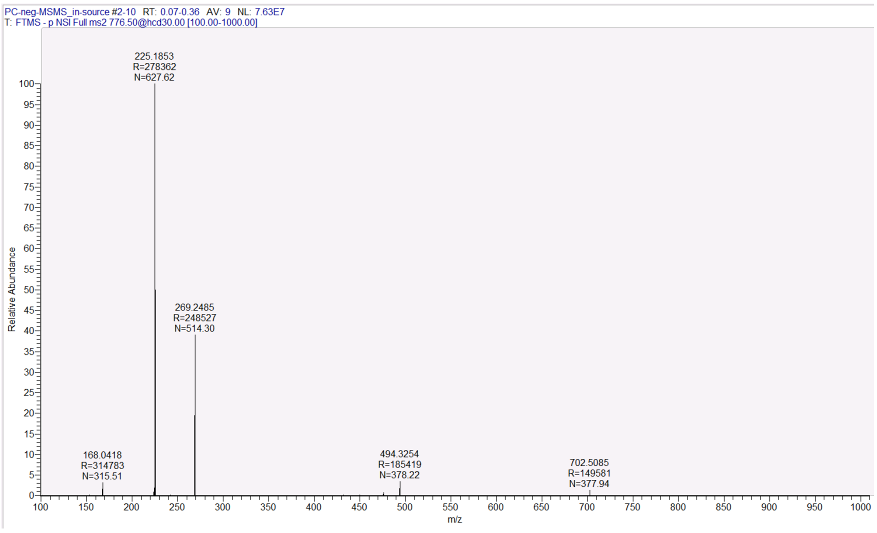

Figure 2: Fragment spectrum of PC 17:0/14:1. Peaks with m/z = 269 and m/z = 225 are FA 17:0 and FA 14:1, respectively. No lyso-PC is formed upon fragmentation.

Another aspect to consider is, that the method applied here is based on nano-ESI with very low flowrates in the nL/min range. Additionally, very volatile solvents are used, which are relatively easy to evaporate during the infusion process. In LC-MS assays, typically much higher flow rates are used and water solvent systems with higher water content. Therefore, in LC-MS higher capillary temperatures are needed in order to evaporate solvents and facilitate ionization, while ionization conditions in nano-ESI are typically less harsh. For example, the capillary temperature in the method used here is 200˚C.

5. Was relative intensity or absolute concentration of lipids used for modelling?

Absolute concentrations in pmol were initially used. However, to work well for predictive modelling the data were "…centered and scaled (Z-score) to avoid predominance of the most abundant features ..." (line 219).
